# Synthesis of new hybrid pyridines catalyzed by Fe_3_O_4_@SiO_2_@urea-riched ligand/Ch-Cl

**DOI:** 10.1038/s41598-023-35849-3

**Published:** 2023-06-10

**Authors:** Narges Zarei, Mohammad Ali Zolfigol, Morteza Torabi, Meysam Yarie

**Affiliations:** Department of Organic Chemistry, Faculty of Chemistry, Bu-Ali Sina University, Hamedan, Iran

**Keywords:** Catalysis, Organic chemistry, Chemical synthesis

## Abstract

Herein, a new heterogeneous catalytic system through modification of urea functionalized magnetic nanoparticles with choline chloride [Fe_3_O_4_@SiO_2_@urea-riched ligand/Ch-Cl] was designed and synthesized. Then, the synthesized Fe_3_O_4_@SiO_2_@urea-riched ligand/Ch-Cl was characterized by using FT-IR spectroscopy, FESEM, TEM, EDS-Mapping, TGA/DTG and VSM techniques. After that, the catalytic usage of Fe_3_O_4_@SiO_2_@urea-riched ligand/Ch-Cl was investigated for the synthesis of hybrid pyridines with sulfonate and/or indole moieties. Delightfully, the outcome was satisfactory and the applied strategy represents several advantages such as short reaction times, convenience of operation and relatively good yields of obtained products. Moreover, the catalytic behavior of several formal homogeneous DESs was investigated for the synthesis of target product. In addition, a cooperative vinylogous anomeric-based oxidation pathway was suggested as rational mechanism for the synthesis of new hybrid pyridines.

## Introduction

**Figure 1:**
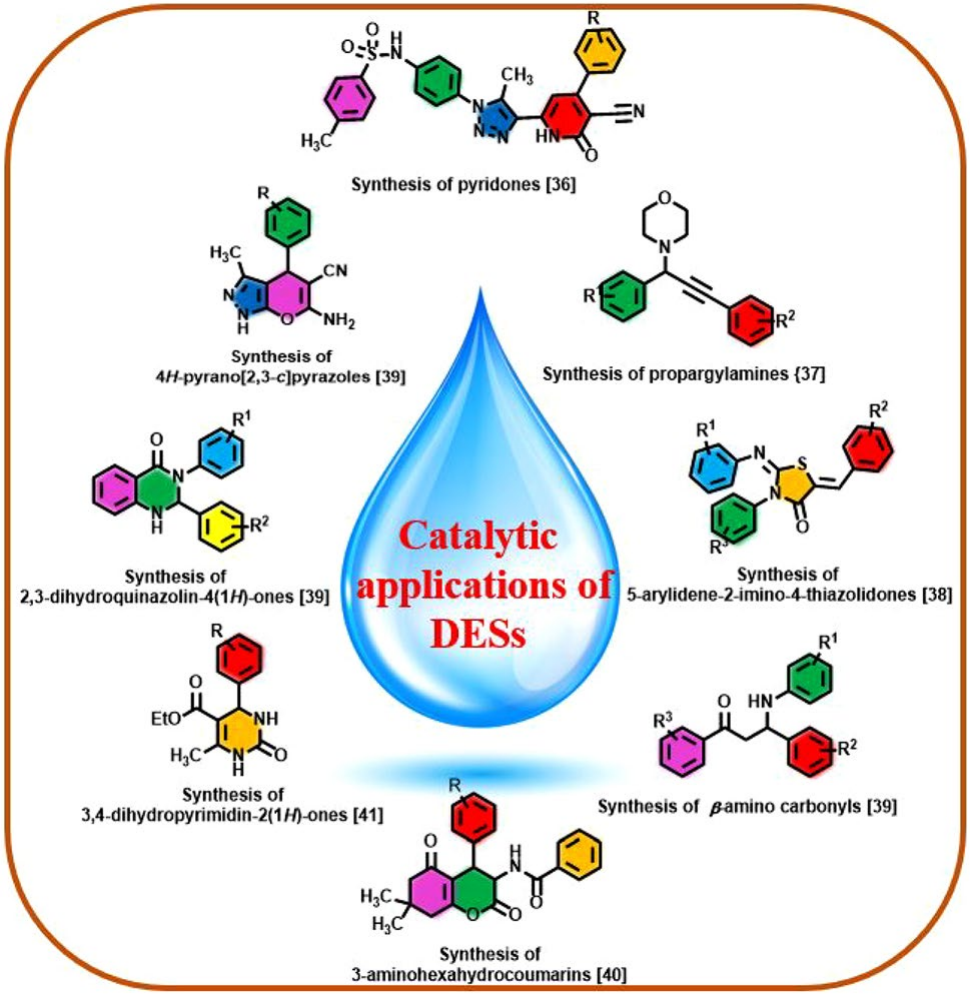
A number of catalytic applications of DESs in multi-component reactions.

Deep eutectic solvents (DESs) as an emerging alternative to ionic liquids and organic solvents have a brilliant breakthrough in many scientific areas^1–4^. Low vapor pressure, tunable physiochemical properties, high polarity, biodegradability, greener and excellent catalytic activity of DESs are usually highlighted^5–8^. Recently, DESs linked to heterogeneous supports, as a new subclass of heterogeneous systems, have a creating and implementing beneficial and neoteric chemical transformations^9–11^. DESs have applied for modification of the surface of some materials such as magnetic nanoparticles (MNPs), metal organic frameworks (MOFs), silicates, covalent organic frameworks (COFs), biopolymers and etc.^12–16^. So, it can be said that these compounds are the forefront of post-synthetic modifications^17^. In this respect, nanomagnetic supported DESs (MDESs) is oriented toward fundamental and synthesis researches. MDESs due to easy reusing and recoverability and workup simplicity create an ingenious insight in many academic and industrial areas^18–21^. These materials have significant potentials and applications in several outlines including solar cells^22^, electrochemistry^23^, redox flow batteries^24^, supercapacitors^25^, biosensors^26^ and chemical separation processes^27^. Also, due to the simultaneous presence of acidic and basic functional groups and response to external magnetic field, MDESs are a prosperous assortment as sustainable media as well as catalysts in many of organic transformations such as coupling reactions^28–30^ and multi-component reactions^31,32^. Recently, there are several studies from catalytic applications of MDESs in multi-component reactions^33–35^. A number of catalytic applications of DESs in multi-component reactions are sketched in below (Fig. 1)^33,36–40^.

Despite extensive research on magnetic nanoparticles, these particles are still being researched by scientists due to their unique features and capabilities. These particles have the ability to be a suitable substrate for connecting organic groups. Magnetic nanoparticles have unique properties such as low toxicity, low cost, high magnetic property, compatibility with the environment, high surface area, chemical stability^41–43^.

These particles are used as sensors, absorption of organic substances (paints, pharmaceuticals, etc.), water disinfection, wastewater treatment, as well as in the field of biomedicine and cancer treatment^44,45^.

Another prominent application of magnetic nanoparticles is their usage as catalysts, which attracted a lot of attention among scientists due to their availability, easy recovery, and reusability^46,47^. Photocatalysis, oxidation/reduction reactions, multi-component reactions, photoelectrochemical catalysis, coupling reactions and chiral catalysis are examples of catalytic applications of MNPs^48–50^. Therefore, immobilizing catalytically active species on magnetic substrates while maintaining or improving their activity also allows for easy separation. For this reason, these catalytic systems are superior to homogeneous systems.

**Figure 2:**
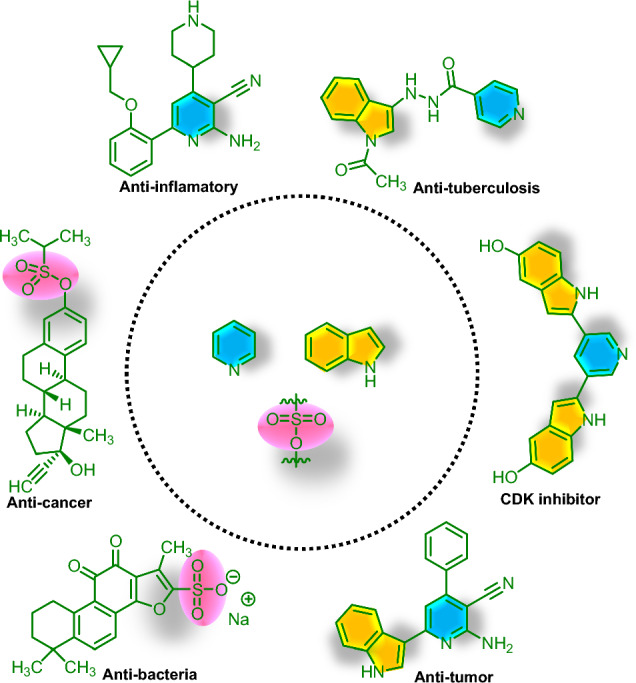
A number of biologically active hybrid molecules.

Molecular hybridization due to its compositional characterization is an interesting structural modification approach which are including of the incorporation of two or more pharmacophores into a single molecule^51,52^. The unique performance of hybrid heterocycles is based on the recognition of pharmacophoric moieties in two or more biologically active molecules which preserved or promoted pre-selected properties of the original templates^52,53^. Hybrid pyridines as distinguished scaffolds of heterocyclic compounds not only are extensively present in pharmaceutical active molecules, agricultural compounds and functional materials^54–58^ but also are one of the most top-selling drugs and can serve as treatment of Alzheimer’s diseases^59^, anticancer^60^ and antihypertensive^61^. Intriguingly, hybrid molecules bearing pyridine, indole and sulfonate moieties have exceptional potential in pharmaceutical and medicinal chemistry such as antioxidant, antiapoptosis, antidyslipidemia, antitumorigenic, antiinflammatory and can enhance the stability and solubility of drugs^62–67^. A number of biologically active hybrid molecules bearing pyridine, indole and sulfonate moieties are sketched in Fig. 2.

**Figure 3:**
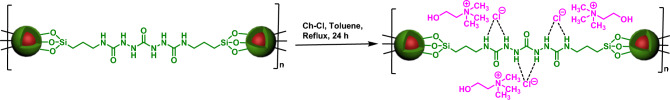
General procedure for the synthesis of Fe_3_O_4_@SiO_2_@urea-riched ligand/Ch-Cl.

**Figure 4:**
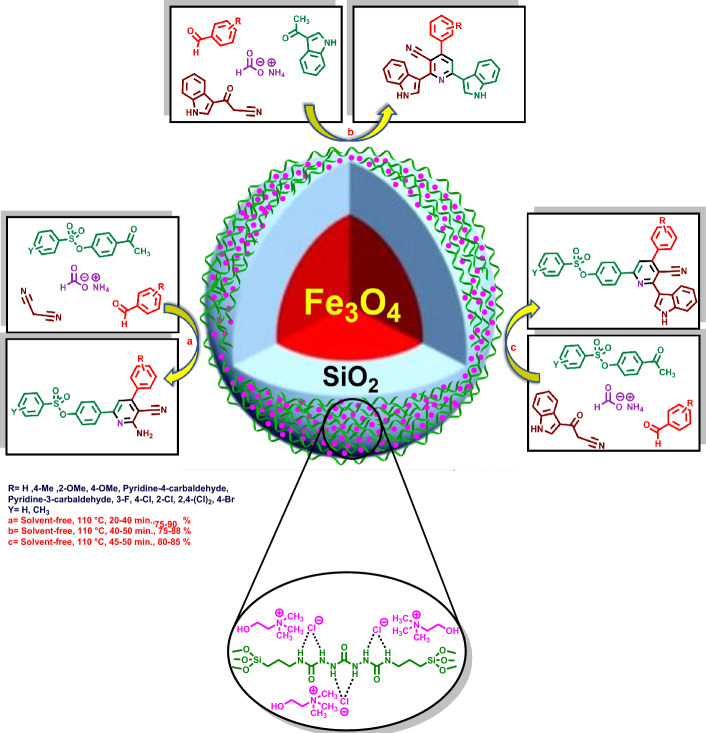
Synthesis of hybrid pyridines bearing indole and/or sulfonate moieties by using Fe_3_O_4_@SiO_2_@urea-riched ligand/Ch-Cl as catalyst.

In this investigation, after synthesis of Fe_3_O_4_@SiO_2_@urea-riched ligand/Ch-Cl, pyridines with indole and/or sulfonate moieties were synthesized in the presence of Fe_3_O_4_@SiO_2_@urea-riched ligand/Ch-Cl via a multi-component reaction strategy^68,69^ (Figs. 3, 4). Also, a cooperative vinylogous anomeric-based oxidation pathway was suggested as plausible mechanism for the synthesis of new hybrid pyridines^70–84^.

## Results and discussion

A literature survey shows that in heterocyclic chemistry as a significant category of organic chemistry, pyridine plays as same as benzene in the concept of aromaticity. On the other hand, hybrid pyridines are the most heterocycle molecules which have been used for various purposes such as medicinal drugs, agricultural adducts, dyes, polymers etc.^85–88^. Therefore, development of hybrid pyridines is one of our main research interests. With this aim, herein we wish to report a new catalytic system for preparation of new hybrid pyridines with aryl, indole and sulfonate moieties.

**Figure 5:**
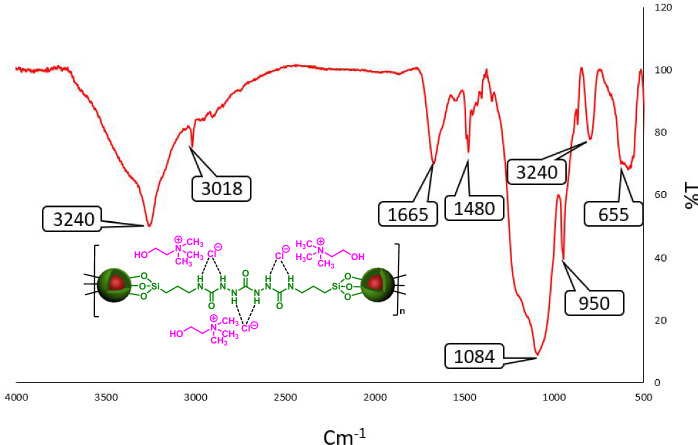
FT-IR spectrum of Fe_3_O_4_@SiO_2_@urea-riched ligand /Ch-Cl.

After synthesis of Fe_3_O_4_@SiO_2_@urea-riched ligand/Ch-Cl, we focused on the precise characterization of its structure. Firstly, FT-IR spectrum of catalyst were investigated (Fig. 5). According to FT-IR spectrum of Fe_3_O_4_@SiO_2_@urea-riched ligand/Ch-Cl, the clear peak of C=O is appeared at 1665 cm^−1^. The vibrational modes of Fe–O, Si–O and NH groups are respectively shown at 655, 1084 and 3240 cm^−1^. Moreover, the broad peak about of 3200 cm^−1^ confirmed the existence of hydroxy group of Ch-Cl and free hydroxy groups in the surface of Fe_3_O_4_.

**Figure 6:**
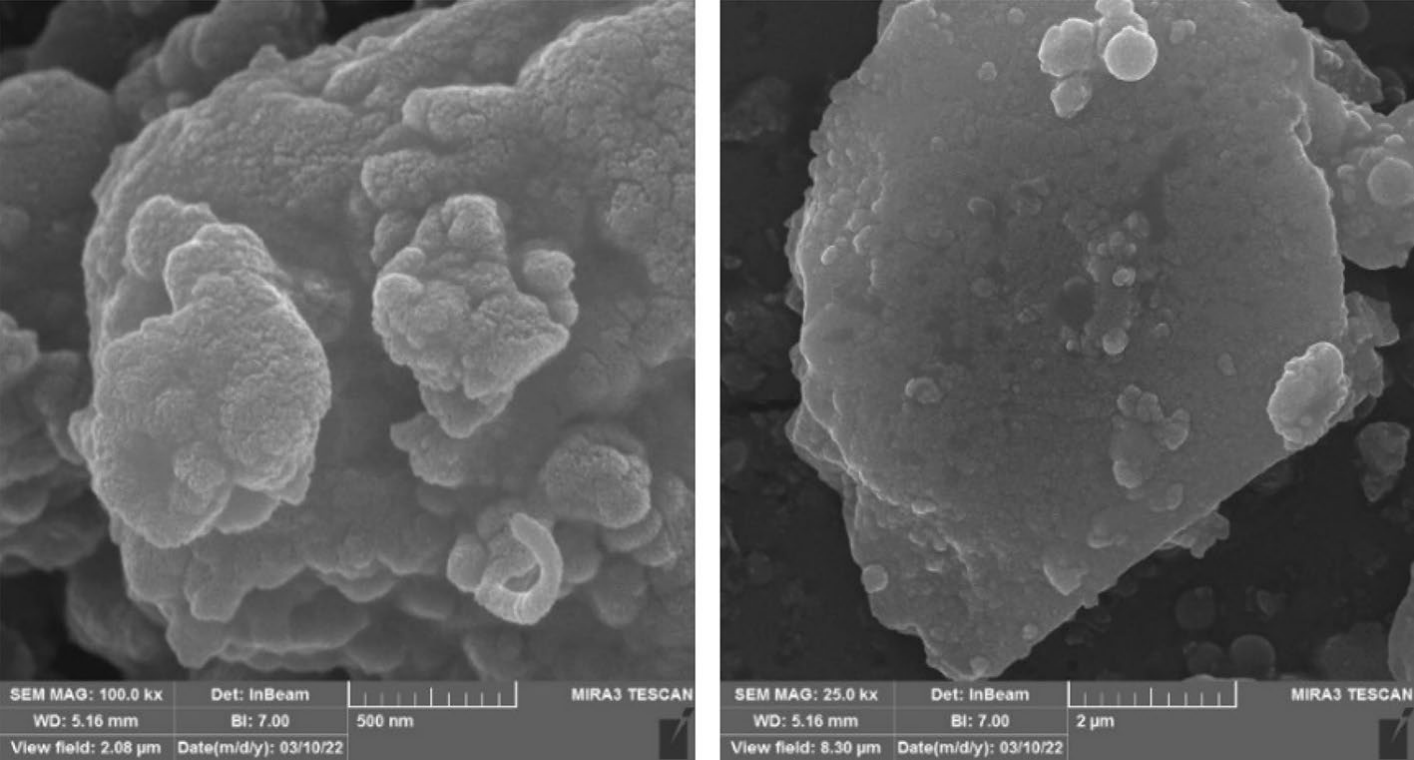
FESEM images Fe_3_O_4_@SiO_2_@urea-riched ligand/Ch-Cl.

**Figure 7:**
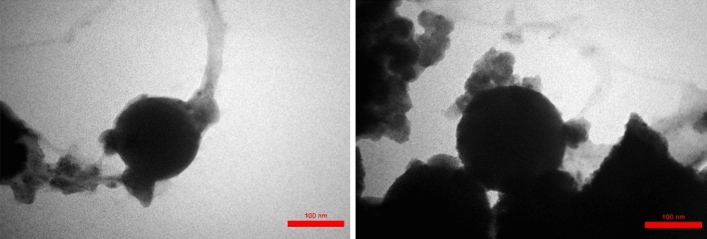
TEM images of Fe_3_O_4_@SiO_2_@urea-riched ligand/Ch-Cl.

Field emission scanning electron microscopy (FESEM) analysis was recorded to check the morphology of Fe_3_O_4_@SiO_2_@urea-riched ligand /Ch-Cl. According to relevant images (Fig. 6), catalyst has a spherical and uniform shapes and its size is in the range of nanometers. Also, TEM analysis was investigated to confirm the formation of the catalyst with spherical morphology and the presence of organic layers on the surface of magnetic nanoparticles is well confirmed (Fig. 7).

**Figure 8:**
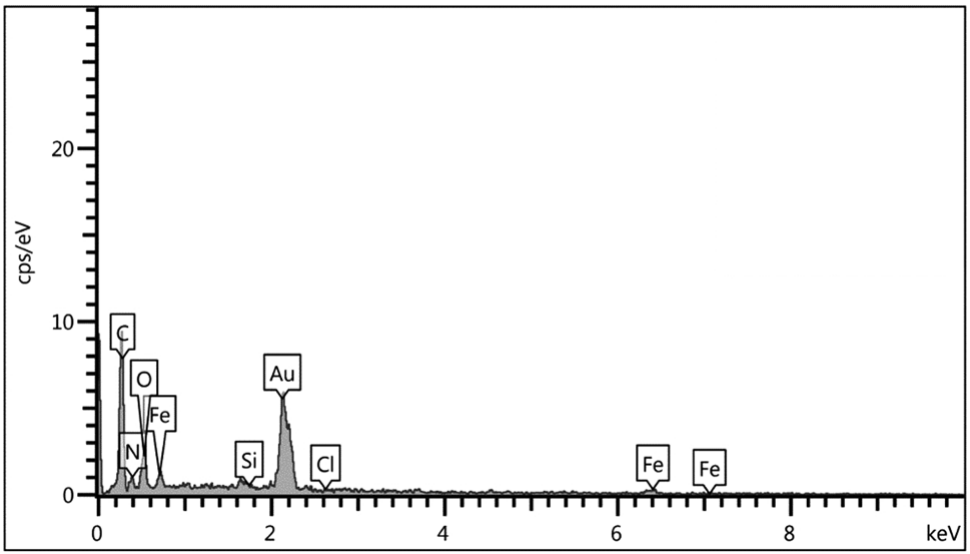
EDS analysis diagram of Fe_3_O_4_@SiO_2_@urea-riched ligand/Ch-Cl.

**Figure 9:**
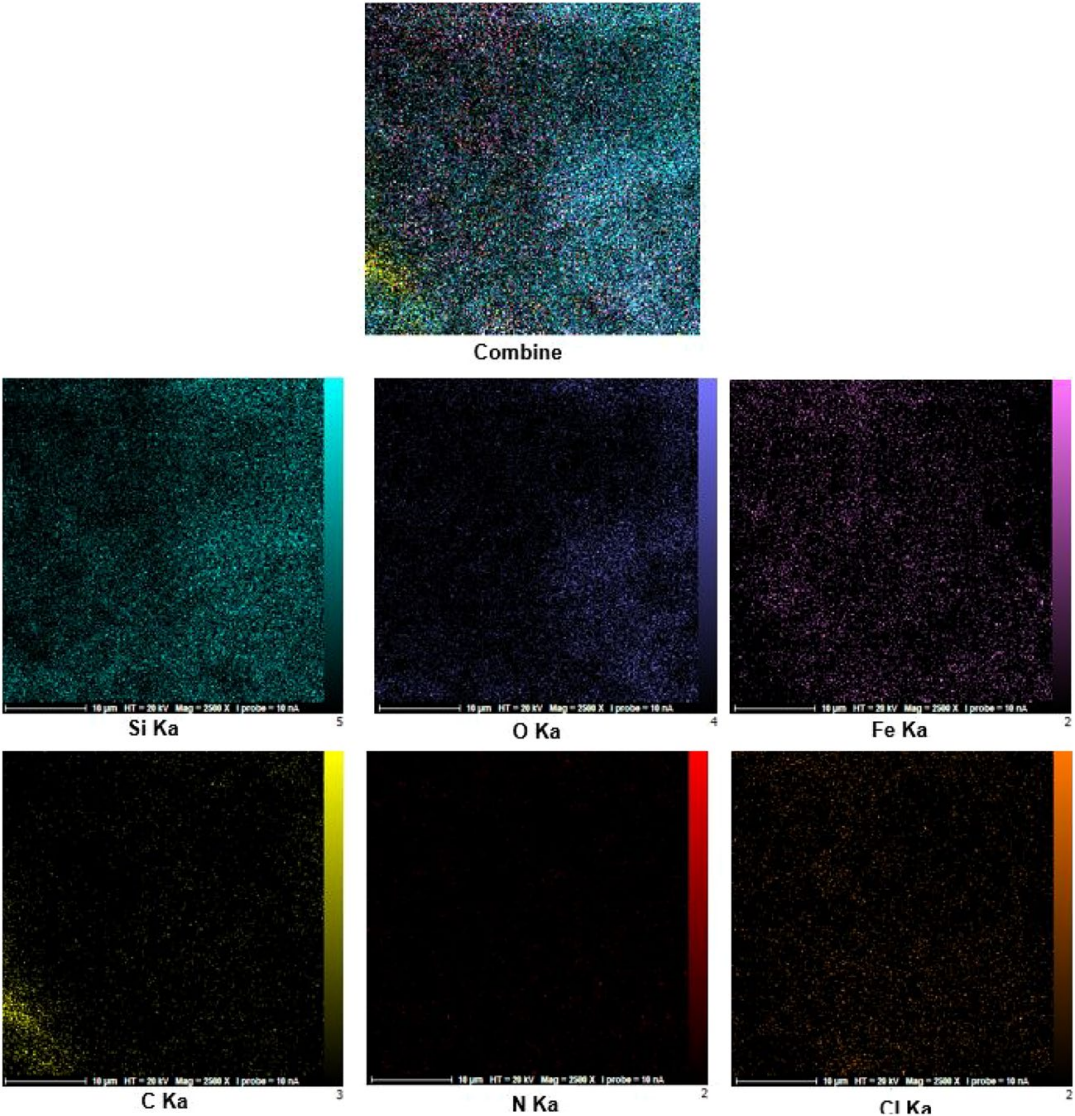
Elemental mapping analysis of Fe_3_O_4_@SiO_2_@urea-riched ligand/Ch-Cl.

Energy-dispersive X-ray spectroscopy (EDS) analysis was used for the examination of expected elements within the catalyst structure. As predicted, the elements of C, N, O, Fe, Cl and Si are presented in the structure of desired catalyst (Fig. 8). In addition, elemental mapping analysis shows how the elements are dispersed and confirmed the existence of abovementioned expected elements in the structure of catalyst (Fig. 9).

**Figure 10:**
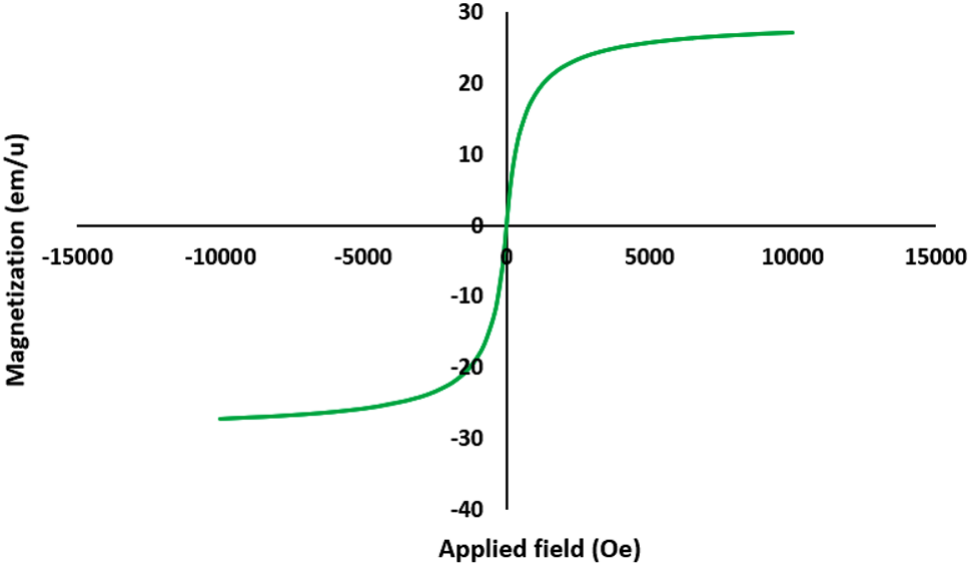
Magnetization curves of Fe_3_O_4_@SiO_2_@urea-riched ligand/Ch-Cl.

For investigation of magnetic properties of target catalyst, VSM technique was performed for Fe_3_O_4_@SiO_2_@urea-riched ligand/Ch-Cl. According to revealed results, the saturation magnetization of Fe_3_O_4_@SiO_2_@urea-riched ligand/Ch-Cl is about 27 emu/g which is enough for the easy separation of the catalyst from the reaction mixture (Fig. 10).

**Figure 11:**
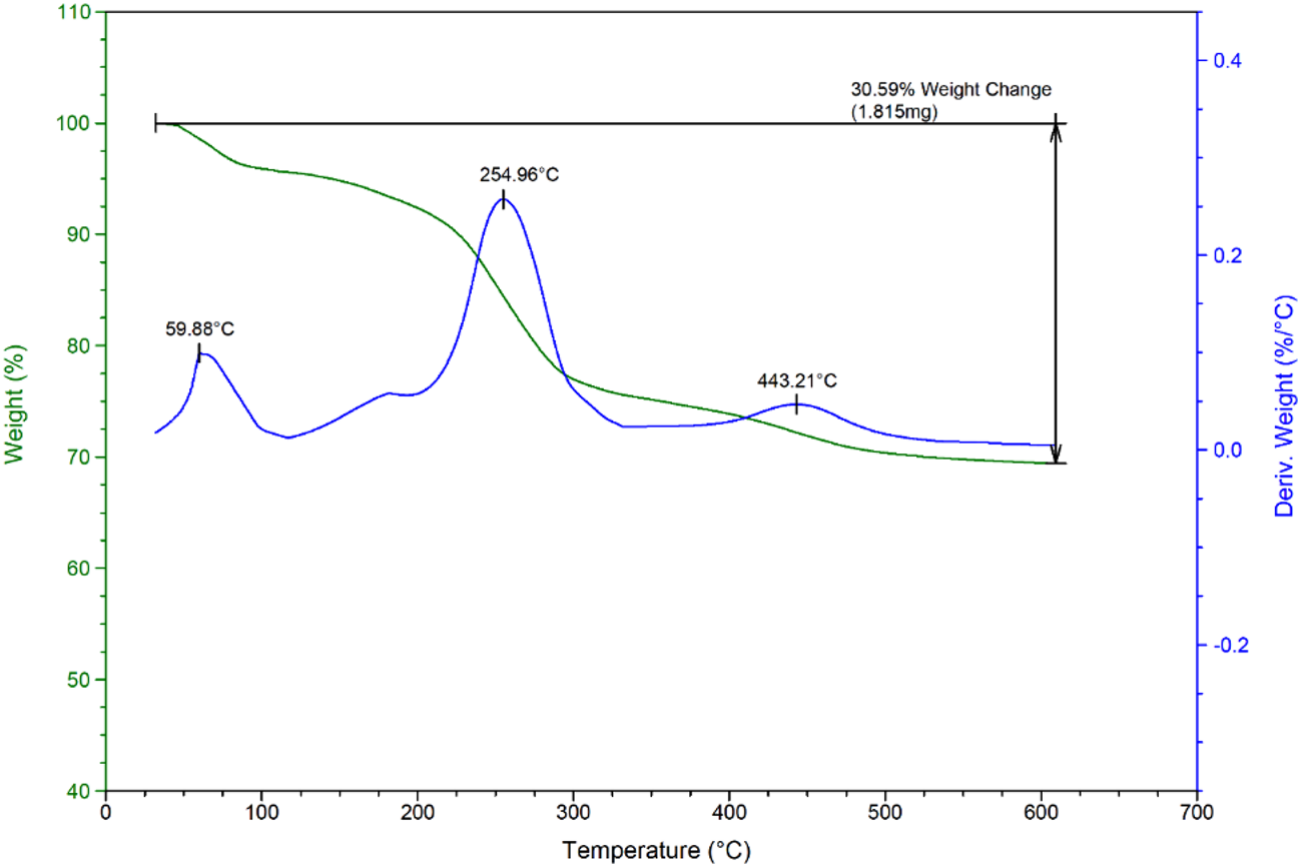
TGA/DTG curves of Fe_3_O_4_@SiO_2_@urea-riched ligand/Ch-Cl.

Thermal stability is another important factor for MDES systems. Therefore, we investigated the thermal stability of the catalyst by using TGA/DTG analysis (Fig. 11). When the catalyst is exposed to the thermal conditions up to 600 °C, two main weight losses are observed at temperatures 255 and 443 °C. The poor weight losses below 110 °C is related to removing of trapped solvents and the significant weight losses at 255 °C is related to decomposition of organic layers. Therefore, it can be said that the catalyst is thermally stable up to this temperature. In an overview, the decrease in the weight of the catalyst by 30.59% indicates the presence of a significant amount of organic ligand on the Fe_3_O_4_ surface.

At the outset of the synthesis of hybrid pyridines, benzaldehyde, 4-acetylphenyl 4-methylbenzenesulfonate, malononitrile and ammonium format were chosen as model substrates for multi-component synthesis of pyridine derivatives. At the first, the reaction conducted in the presence of different amounts of catalyst such as 5, 10 and 20 mg. Also, the model reaction was tested in the absence of any catalyst. Anyway, the best result was obtained by using 10 mg of catalyst. Subsequent study on the effect of the temperature parameter displayed that 110 °C is the most suitable temperature to provide activation energy for the model reaction. After that, for the investigation of solvent effect, the model reaction was performed in several formal polar and nonpolar solvents and also, solvent free conditions. Nonetheless, due to high yield and low toxicity of reaction and simplicity of work up, solvent free conditions were chosen as proper conditions. More details are given in the Table 1. The bold values indicates the optimal reaction conditions.

**Table 1:** Optimization of reaction conditions for synthesis of **1a**^**a**^.


Entry	Solvent	Temperature (°C)	Catalyst loading (mg)	Time (min.)	Yield (%)^b^
**1**^**c**^	**–**	**110**	**10**	**30**	**75**
2	**–**	110	20	40	60
3	**–**	110	5	70	50
4	**–**	110	**–**	60	20
5	**–**	110	**–**	120	38
6	**–**	100	10	50	80
7	**–**	90	10	70	70
8	**–**	80	10	100	40
9	**–**	70	10	180	35
10	H_2_O	Reflux	10	60	**–**
11	EtOH	Reflux	10	60	50
12	*n*-Hexane	Reflux	10	60	**–**
13	EtOAc	Reflux	10	60	**–**
14	CHCl_3_	Reflux	10	60	**–**

^a^Reaction conditions: 4-acetylphenyl 4-methylbenzenesulfonate (1 mmol, 0.290 g), malononitrile (1 mmol, 0.066 g), ammonium format (1.5 mmol, 0.094 g), benzaldehyde (1 mmol, 0.106 g), ^b^Isolated yields. ^c^Optimal data.

Significant values are given in bold.

In a separate study, the model reaction was performed in the presence of formal homogeneous DESs. For this purpose, several selected homogeneous DESs was prepared^89–94^ and were used as catalyst for the model reaction. Distinguishingly, these materials have a good response to the synthesis of target molecule and all of products have a relatively good yield (Table 2). Nevertheless, devoid of suitable recycling and reusing of the catalyst in homogeneous systems is one of the defecting of these systems, while Fe_3_O_4_@SiO_2_@urea-riched ligand/Ch-Cl as heterogeneous catalyst can easily recycled and reused.

**Table 2:** Investigation of catalytic performance of selected homogeneous DESs upon model reaction for **1a**^a^.

Entry	Catalyst	The amount of catalyst	Yield (%)
1	Fe_3_O_4_@SiO_2_@urea-riched ligand/Ch-Cl	10 mg	75
2	Ch-Cl/urea	10 mol%	77
3	Ch-Cl/thiourea	10 mol%	66
4	Ch-Cl/Citric acid	10 mol%	70
5	Ch-Cl/Benzamide	10 mol%	60
6	Ch-Cl/ Ascorbic acid	10 mol%	65
7	Ch-Cl/ Benzoic acid	10 mol%	70
8	Ch-Cl/Acetamide	10 mol%	60
9	Ch-Cl/ Glycerol	10 mol%	55

^a^Reaction conditions: 4-acetylphenyl 4-methylbenzenesulfonate (1 mmol, 0.290 g), malononitrile (1 mmol, 0.066 g), ammonium format (1.5 mmol, 0.094 g), benzaldehyde (1 mmol, 0.106 g), solvent-free, 110 °C, 30 min.

For the validation of the importance of target catalyst, the model reaction was also performed in the presence of relative intermediates of Fe_3_O_4_@SiO_2_@urea-riched ligand/Ch-Cl and some of formal catalysts such as Lewis acids, protic acids, hydrogen bond and basic catalysts. Using Fe_3_O_4_@SiO_2_@urea-riched ligand/Ch-Cl as catalyst gave the best yield. It goes without saying that using of urea, thiourea and K_2_CO_3_ as catalyst have relatively good yield. Nevertheless, these catalysts do not have the ability to recycle and reuse, which are the basic capabilities of a complete catalyst. (Table 3).

**Table 3:** Comparative investigation of catalytic performance of Fe_3_O_4_@SiO_2_@urea-riched ligand/Ch-Cl and its relative intermediates as well as some known catalysts upon model reaction for **1a**^a^.

Entry	Catalyst	The amount of catalyst	Yield (%)
1	Fe_3_O_4_	10 mg	40
2	Fe_3_O_4_@SiO_2_	10 mg	40
3	Fe_3_O_4_@SiO_2_@urea-riched ligand	10 mg	70
4	Fe_3_O_4_@SiO_2_@urea-riched ligand/Ch-Cl	10 mg	75
5	Ch-Cl	10 mol%	50
6	FeCl_3_	10 mol%	50
7	Al(HSO_4_)_3_	10 mol%	45
8	Ca(HSO_4_)_2_	10 mol%	35
9	Zn(HSO_4_)_2_	10 mol%	35
10	Fe(HSO_4_)_3_	10 mol%	40
11	NH_2_SO_3_H	10 mol%	45
12	H_3_BO_3_	10 mol%	40
13	Urea	10 mol%	75
14	Thiourea	10 mol%	60
15	K_2_CO_3_	10 mol%	70
16	NaOH	10 mol%	Trace
17	C_5_H_5_N	10 mol%	45

^a^Reaction conditions: 4-acetylphenyl 4-methylbenzenesulfonate (1 mmol, 0.290 g), malononitrile (1 mmol, 0.066 g), ammonium format (1.5 mmol, 0.094 g), benzaldehyde (1 mmol, 0.106 g), solvent-free, 110 °C, 30 min.

In a comparative and precise study, for the investigation of the ability of ammonium format as reagent, we used several ammonium sources including ammonium format, ammonium acetate, ammonium sulfate, ammonium carbonate, ammonium florid, ammonium dichromate, ammonium chloride and ammonium nitrate upon model reaction. According to revealed results (Table 4), ammonium format revealed better performance for the synthesis of **1a** molecule.

**Table 4:** Comparison of different ammonium sources as reagent upon model reaction for **1a**^a^.

Entry	Reagent	Yield (%)
**1**	**NH**_**4**_**HCO**_**2**_	**75**
2	NH_4_OAc	70
3	(NH_4_)_2_SO_4_	–
4	(NH_4_)_2_CO_3_	60
5	NH_4_F	–
6	(NH_4_)_2_Cr_2_O_7_	50
7	NH_4_Cl	–
8	NH_4_NO_3_	35

^a^Reaction conditions: 4-acetylphenyl 4-methylbenzenesulfonate (1 mmol, 0.290 g), malononitrile (1 mmol, 0.066 g), benzaldehyde (1 mmol, 0.106 g), ammonium source (1.5 mmol), solvent-free, 110 °C, 30 min.

Significant values are given in bold.

Based on the in-hand results of optimization reactions, the generality of the reaction for synthesis of various hybrid pyridines was investigated. For this purpose, variety of aromatic aldehydes with electron-poor or electron-rich aryl groups, three different methyl ketones bearing indole or sulfonate groups and malononitrile or 3-(1*H*-indol-3-yl)-3-oxopropanenitrile were applied for the synthesis of hybrid pyridine derivatives. The tolerance of the reaction to diverse starting materials displayed the broad application scope of the present route in the synthesis of complex hybrid pyridines (Table 5).

**Table 5:**
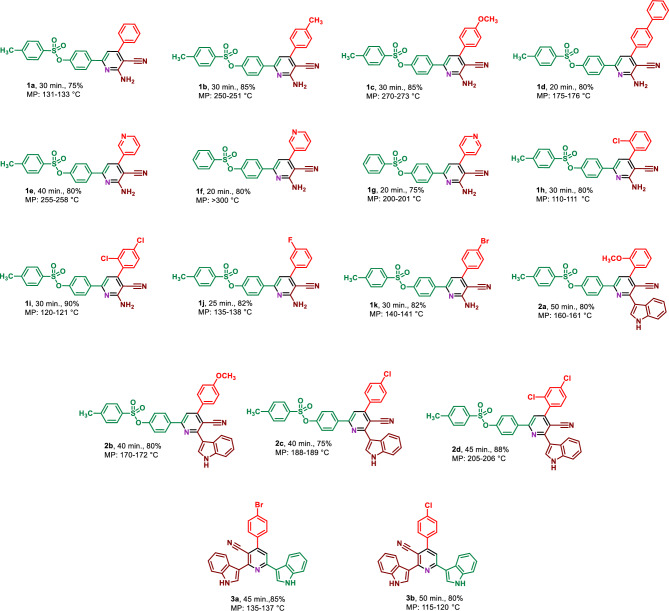
Synthesis of hybrid pyridines with indole and sulfonate moieties by using Fe_3_O_4_@SiO_2_@urea-riched ligand /Ch-Cl^a^.

^a^Reaction conditions: 4-acetylphenyl 4-methylbenzenesulfonate (1 mmol, 0.290 g) or 3-acetyl indole (1 mmol, 0.1590) malononitrile (1 mmol, 0.066 g) or 3-(1*H*-indol-3-yl)-3-oxopropanenitrile (1 mmol, 0.184 g), ammonium format (1.5 mmol, 0.094 g), benzaldehyde (1 mmol, 0.106 g), catalyst (10 mg), solvent-free, 110 °C.

**Figure 12:**
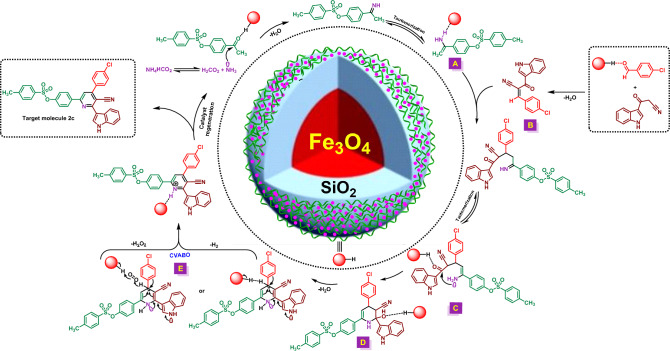
The plausible mechanism for the synthesis of **2c**.

Based on our knowledge from the synthesis of hybrid pyridine rings, we suggested a plausible mechanism for the synthesis of **2c** (Fig. 12). At the first step, the carbonyl functional group of 4-acetylphenyl 4-methylbenzenesulfonate is activated with catalyst and reacted with ammonia (arisen from thermal dissociation of ammonium format) and via a tautomerization process gives intermediate **A**. In another part of the reaction, aldehyde was activated by the catalyst and by a condensation reaction with 3-(1*H*-indol-3-yl)-3-oxopropanenitrile yields Knoevenagel intermediate (**B**). Subsequently, intermediate **A** was reacted with intermediate **B** and then via a tautomerization process, intermediate **C** was formed. Then, intermediate **C** undergoes intramolecular cyclization to gives intermediate **D**. In the next step, this intermediate undergoes a H_2_O removing process which leads the formation of intermediate **E**. Finally, molecular H_2_ (inert conditions) or H_2_O_2_ (air conditions) was released based on a cooperative vinylogous anomeric-based oxidation (CVABO)^70–84^ to yields target molecule **2c**.

**Figure 13:**
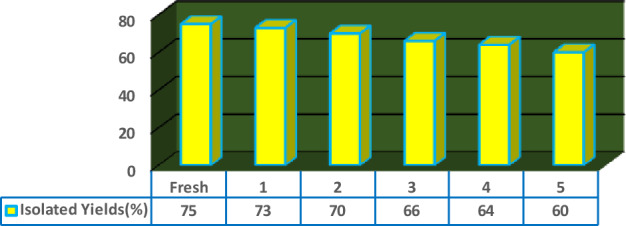
Diagram of recycling and reusing of Fe_3_O_4_@SiO_2_@urea-riched ligand/Ch-Cl in the synthesis of **1a**.

M﻿agnetic substrates serve as ideal systems in recoverable catalysts and investigation of recycling and reusing potential of nanomagnetic catalyst is very important. Therefore, we examined the recycling and reusing ability of Fe_3_O_4_@SiO_2_@urea-riched ligand/Ch-Cl for the synthesis of **1a** which leads to acceptable results. After running and performing each of reactions, the mixture of reaction was dissolved in CH_2_Cl_2_ and insoluble catalyst was separated from the reaction mixture and washed with CH_2_Cl_2_ (3 $\times$ 10 mL) and air dried. This work was conducted five times without significant reduction in yield of the reaction (Fig. 13). In addition, FT-IR spectrum was used for investigation the stability of recovered catalyst (See ESI).

## Experimental section

### Experimental procedure for the synthesis of Fe_3_O_4_@SiO_2_@urea-riched ligand/Ch-Cl

At the first, Fe_3_O_4_@SiO_2_@urea-riched ligand was synthesized according to our previous report^70–76^. Then, in a 100 mL round-bottomed flask 1 g of Fe_3_O_4_@SiO_2_@urea-riched ligand and choline chloride (6 mmol, 0.837 g) and 100 mL of toluene as solvent were added and was refluxed for 24 h. After completing of reaction, the desired catalyst was separated by using external magnet and washed with *n*-hexane and EtOH several times and dried in air condition.

### General experimental route for the synthesis of hybrid pyridine derivatives

In 10 mL round-bottomed flask methyl ketones (1 mmol), aromatic aldehydes (1 mmol), malononitrile (1 mmol, 0.066 g) or 3-(1*H*-indol-3-yl)-3-oxopropanenitrile (1 mmol, 0.184 g), ammonium format (1.5 mmol, 0.094 g) and 10 mg of catalyst were added and the mixture of reaction was stirred at 110 °C for appropriate times as indicated in Table 2. The progress of reactions was monitored by TLC techniques (*n*-hexane/ethylacetate, 6/4). After completing of each reaction, the mixture of reaction was dissolved in CH_2_Cl_2_ and the catalyst was separated from organic mixture. Then, each of products was purified by TLC plate techniques with *n*-hexane/ethyl acetate.

### Spectral data

#### 4-(6-Amino-5-cyano-4-phenylpyridin-2-yl)phenyl 4-methylbenzenesulfonate (1a)

M.P. 131–133 °C, FT-IR (KBr, υ, cm^−1^): 3489, 3367, 2209, 1597, 1493, 1091. ^1^H NMR (250 MHz, DMSO_d6_) δ_ppm_ 8.10 (d, *J* = 10 Hz, 2H, Aromatic), 7.73 (d, *J* = 10 Hz, 2H, Aromatic), 7.65–7.62 (m, 2H, Aromatic), 7.52–7.43 (m, 5H, Aromatic), 7.23 (s, 1H, Aromatic), 7.15–7.03 (m, 4H, Aromatic and NH_2_), 2.39 (s, 3H, CH_3_). ^13^C NMR (76 MHz, DMSO_d6_) δ_ppm_ 161.2, 157.5, 155.5, 150.7, 146.4, 137.0, 130.70, 130.10, 129.40, 129.2, 128.8, 122.7, 117.4, 109.8, 87.4, 21.6. MS (*m*/*z*) = calcd. for C_25_H_19_N_3_O_3_S: 441.5, found: 441.3.

#### 4-(6-Amino-5-cyano-4-(*p*-tolyl)pyridin-2-yl)phenyl 4-methylbenzenesulfonate (1b)

M.P. 250–251 °C, FT-IR (KBr, υ, cm^−1^): 3508, 3391, 2922, 2206, 1367, 1153. ^1^H NMR (301 MHz, DMSO_d6_) δ_ppm_ 8.14 (d, *J* = 9 Hz, 2H, Aromatic), 7.78 (d, *J* = 9 Hz, 2H, Aromatic), 7.59 (d, *J* = 9 Hz, 2H, Aromatic), 7.50 (d, *J* = 9 Hz, 2H, Aromatic), 7.37 (d, *J* = 6 Hz, 2H, Aromatic), 7.25 (s, 1H, Aromatic), 7.15 (d, *J* = 6 Hz, 2H, Aromatic), 7.04 (s, 2H, NH_2_), 2.43 (s, 3H, CH_3_), 2.41 (s, 3H, CH_3_). ^13^C NMR (76 MHz, DMSO_d6_) δ_ppm_ 161.3, 157.5, 155.5, 150.7, 146.4, 140.0, 137.1, 134.4, 131.7, 130.8, 129.8, 129.4, 128.8, 128.7, 122.7, 117.5, 109.7, 87.3, 21.7, 21.4. MS (*m/z*) = calcd. for C_26_H_21_N_3_O_3_S: 455.5, found: 455.2.

#### 4-(6-Amino-5-cyano-4-(4-methoxyphenyl)pyridin-2-yl)phenyl 4-methylbenzenesulfonate (1c)

M.P. 270–273 °C, FT-IR (KBr, υ, cm^−1^): 3447, 3390, 2925, 2206, 1608, 1515, 1368, 1153. ^1^H NMR (301 MHz, DMSO_d6_) δ_ppm_ 8.14 (d, *J* = 9 Hz, 2H, Aromatic), 7.79 (d, *J* = 9 Hz, 2H, Aromatic), 7.67 (d, *J* = 9 Hz, 2H, Aromatic), 7.50 (d, *J* = 9 Hz, 2H, Aromatic), 7.26 (s, 1H, Aromatic), 7.17 (s, 1H, Aromatic), 7.14 (s, 2H, Aromatic), 7.11 (s, 1H, Aromatic), 7.01 (s, 2H, NH_2_), 3.86 (s, 3H, OCH_3_), 2.44 (s, 3H, CH_3_). ^13^C NMR (76 MHz, DMSO_d6_) δ_ppm_ 161.4, 161.0, 157.4, 155.1, 150.6, 146.4, 140.9, 137.2, 131.7, 130.8, 130.4, 129.4, 128.8, 122.7, 117.7, 114.6, 109.6, 87.2, 55.9, 21.7. MS (*m*/*z*) = calcd. for C_26_H_21_N_3_O_4_S: 471.5, found: 471.2.

#### 4-(4-([1,1'-Biphenyl]-4-yl)-6-amino-5-cyanopyridin-2-yl)phenyl 4-methylbenzenesulfonate (1d)

M.P. 175–176 °C, FT-IR (KBr, υ, cm^−1^): 3502, 3402, 2205, 1366, 1154. ^1^H NMR (301 MHz, DMSO_d6_) δ_ppm_ 8.17 (d, *J* = 9 Hz, 2H, Aromatic), 7.88 (d, *J* = 9 Hz, 2H, Aromatic), 7.81–7.77 (m, 5H, Aromatic), 7.56–7.44 (m, 5H, Aromatic), 7.35 (s, 1H, Aromatic), 7.17 (d, *J* = 9 Hz, 2H), 7.10 (s, 2H, NH_2_), 2.44 (s, 3H, CH_3_). ^13^C NMR (76 MHz, DMSO_d6_) δ_ppm_ 161.3, 157.6, 155.1, 150.7, 146.5, 141.9, 139.7, 137.1, 136.3, 131.7, 130.8, 129.6, 129.5, 129.5, 128.8, 128.5, 127.4, 127.3, 122.7, 117.5, 109.8, 87.3, 21.7. MS (*m/z*) = calcd. for C_31_H_23_N_3_O_3_S: 517.6, found: 517.3.

#### 4-(6'-Amino-5'-cyano-[3,4'-bipyridin]-2'-yl)phenyl 4-methylbenzenesulfonate (1e)

M.P. 255–258 °C, FT-IR (KBr, υ, cm^−1^): 3442, 3373, 2925, 2213, 1369, 1153. ^1^H NMR (301 MHz, DMSO_d6_) δ_ppm_ 8.88 (dd, *J* = 3, 0.8 Hz, 1H), 8.75 (dd, *J* = 6, 3 Hz, 1H), 8.17 (m, 2H), 8.12 (m, 1H, Aromatic), 7.97 (s, 1H, Aromatic), 7.79 (d, *J* = 9 Hz, 2H, Aromatic), 7.61 (ddd, *J* = 7.9, 4.8, 0.9 Hz, 1H, Aromatic), 7.50 (d, *J* = 6 Hz, 2H), 7.38 (s, 1H, Aromatic), 7.18 (s, 2H, NH_2_), 7.15 (s, 1H, Aromatic), 2.44 (s, 3H, CH_3_). MS (*m/z*) = calcd. for C_24_H_18_N_4_O_3_S: 442.5, found: 442.2.

#### 4-(6'-Amino-5'-cyano-[3,4'-bipyridin]-2'-yl)phenyl benzenesulfonate (1f)

M.P. > 300 °C, FT-IR (KBr, υ, cm^−1^): 3426, 3317, 3168, 2212, 1642, 1448, 1354, 1152. ^1^H NMR (250 MHz, DMSO_d6_) δ_ppm_ 8.84–8.80 (m, 1H, Aromatic), 8.70 (d, *J* = 5 Hz, 1H, Aromatic), 8.13 (d, *J* = 7.5 Hz, 2H, Aromatic), 7.92–7.79 (m, 3H, Aromatic), 7.69–7.66 (m, 3H, Aromatic), 7.60–7.54 (m, 1H, Aromatic), 7.34 (s, 1H, Aromatic), 7.20–7.11 (m, 4H, Aromatic and NH_2_). ^13^C NMR (76 MHz, DMSO_d6_) δ_ppm_ 161.0, 157.8, 151.0, 149.1, 136.6, 136.5, 135.6, 132.1, 130.8, 130.3, 129.5, 128.7, 124.0, 122.7, 119.1, 117.1, 116.1, 109.9, 87.4. MS (*m/z*) = calcd. for C_23_H_16_N_4_O_3_S: 428.5, found: 428.3.

#### 4-(6-Amino-5-cyano-[4,4'-bipyridin]-2-yl)phenylbenzenesulfonate (1g)

M.P. 200–201 °C, FT-IR (KBr, υ, cm^−1^): 3442, 3291, 3170, 2216, 1375, 1153. ^1^H NMR (301 MHz, DMSO) δ_ppm_ 8.75 (d, *J* = 6 Hz, 2H, Aromatic), 7.94 (dd, *J* = 6, 1.3 Hz, 2H, Aromatic), 7.74–7.65 (m, 8H, Aromatic), 7.23 (d, *J* = 9 Hz, 2H, Aromatic), 7.03 (s, 2H, NH_2_). ^13^C NMR (76 MHz, DMSO_d6_) δ_ppm_ 154.5, 150.5, 150.2, 149.2, 147.6, 145.2, 136.8, 135.7, 134.8, 131.1, 130.5, 128.7, 123.7, 122.8, 118.7, 95.6. MS (*m/z*) = calcd. for C_23_H_16_N_4_O_3_S: 428.5, found: 428.4.

#### 4-(6-Amino-4-(2-chlorophenyl)-5-cyanopyridin-2-yl)phenyl 4-methylbenzenesulfonate (1h)

M.P. 110–111 °C, FT-IR (KBr, υ, cm^−1^): 3489, 3373, 3168, 2918, 2213, 1370, 1153. ^1^H NMR (301 MHz, DMSO_d6_) δ_ppm_ 8.13 (d, *J* = 9 Hz, 2H, Aromatic), 7.77 (d, *J* = 6 Hz, 2H, Aromatic), 7.68–7.65 (m, 1H, Aromatic), 7.53–7.48 (m, 5H, Aromatic), 7.23 (s, 1H, Aromatic), 7.20–7.13 (m, 4H, Aromatic and NH_2_), 2.43 (s, 3H, CH_3_). ^13^C NMR (76 MHz, DMSO_d6_) δ_ppm_ 160.1, 157.5, 153.8, 150.8, 146.5, 136.8, 136.5, 131.7, 131.4, 131.1, 130.8, 130.2, 129.4, 129.1, 128.8, 128.0, 122.8, 116.4, 110.3, 89.3, 21.7. MS (*m/z*) = calcd. for C_25_H_18_ClN_3_O_3_S: 475.9, found: 475.2.

#### 4-(6-Amino-5-cyano-4-(2,4-dichlorophenyl)pyridin-2-yl)phenyl 4-methylbenzenesulfonate (1i)

M.P. 120–121 °C, FT-IR (KBr, υ, cm^−1^): 3485, 3376, 2925, 2213, 1502, 1447, 1371, 1153. ^1^H NMR (301 MHz, DMSO_d6_) δ_ppm_ 8.12 (d, *J* = 9 Hz, 2H, Aromatic), 7.88 (d, *J* = 1.9 Hz, 1H, Aromatic), 7.77 (d, *J* = 6 Hz, 2H, Aromatic), 7.63 (dd, *J* = 9, 3 Hz, 1H, Aromatic), 7.57 (s, 1H, Aromatic), 7.50 (d, *J* = 9 Hz, 2H, Aromatic), 7.25 (s, 1H, Aromatic), 7.21 (s, 2H, NH_2_), 7.15 (d, *J* = 9 Hz, 2H, Aromatic), 2.43 (s, 3H, CH_3_). ^13^C NMR (76 MHz, DMSO_d6_) δ_ppm_ 160.5, 157.7, 152.7, 150.8, 146.5, 136.7, 135.5, 135.3, 133.0, 132.5, 131.7, 130.8, 129.7, 129.4, 128.8, 128.3, 122.8, 116.3, 110.2, 89.1, 21.7. MS (*m/z*) = calcd. for C_25_H_17_Cl_2_N_3_O_3_S: 510.4, found: 510.3.

#### 4-(6-Amino-5-cyano-4-(3-fluorophenyl)pyridin-2-yl)phenyl 4-methylbenzenesulfonate (1j)

M.P. 135–138 °C, FT-IR (KBr, υ, cm^−1^): 3475, 3382, 2211, 1615, 1447, 1369, 1153. ^1^H NMR (301 MHz, DMSO_d6_) δ_ppm_ 8.16 (d, *J* = 9 Hz, 2H, Aromatic), 7.79 (d, *J* = 9 Hz, 2H, Aromatic), 7.59 (s, 1H, Aromatic), 7.55 (s, 1H, Aromatic), 7.51–7.49 (m, 3H, Aromatic), 7.43–7.37 (m, 1H, Aromatic), 7.32 (s, 1H, Aromatic), 7.17 (s, 1H, Aromatic), 7.14–7.13 (m, 3H, Aromatic and NH_2_), 2.44 (s, 3H, CH_3_). ^13^C NMR (76 MHz, DMSO_d6_) δ_ppm_ 164.1, 161.2, 157.8, 154.1, 150.8, 146.5, 139.5, 139.4, 136.9, 131.7, 130.8, 129.5, 128.8, 125.1, 122.7, 117.2, 116.8, 116.1, 115.7, 109.9, 87.4, 21.7. MS (*m/z*) = calcd. for C_25_H_18_FN_3_O_3_S: 459.5, found: 459.2.

#### 4-(6-Amino-4-(4-bromophenyl)-5-cyanopyridin-2-yl)phenyl 4-methylbenzenesulfonate (1k)

M.P. 140–141 °C, FT-IR (KBr, υ, cm^−1^): 3479, 3380, 2923, 2213, 1594, 1492, 1370, 1153. ^1^H NMR (301 MHz, DMSO_d6_) δ_ppm_ 8.14 (d, *J* = 9 Hz, 2H, Aromatic), 7.78 (d, *J* = 6 Hz, 4H, Aromatic), 7.65 (d, *J* = 9 Hz, 2H, Aromatic), 7.50 (d, *J* = 9 Hz, 2H, Aromatic), 7.29 (s, 1H, Aromatic), 7.17–7.12 (m, 4H, Aromatic, NH_2_), 2.44 (s, 3H, CH_3_). ^13^C NMR (76 MHz, DMSO_d6_) δ_ppm_ 161.2, 157.7, 154.4, 150.7, 146.5, 137.0, 136.4, 132.2, 131.7, 131.0, 130.8, 129.5, 128.8, 123.8, 122.7, 117.2, 116.2, 109.7, 87.2, 21.7. MS (*m*/*z*) = calcd. for C_25_H_18_BrN_3_O_3_S: 520.4, found: 521.1.

#### 4-(5-Cyano-6-(1*H*-indol-3-yl)-4-(2-methoxyphenyl)pyridin-2-yl)phenyl 4-methylbenzenesulfonate (2a)

M.P. 160–161 °C, FT-IR (KBr, υ, cm^−1^): 3396, 2928, 2223, 1374, 1168. ^1^H NMR (301 MHz, DMSO_d6_) δ_ppm_ 11.92 (s, 1H, NH), 8.34 (d, *J* = 9 Hz, 4H, Aromatic), 7.90 (s, 1H, Aromatic), 7.82 (d, *J* = 9 Hz, 2H, Aromatic), 7.59–7.56 (m, 2H, Aromatic), 7.54–7.48 (m, 3H, Aromatic), 7.28– 7.23 (m, 5H, Aromatic), 7.17 (t, *J* = 9 Hz, 1H, Aromatic), 3.85 (s, 3H, OCH_3_), 2.44 (s, 3H, CH_3_). ^13^C NMR (76 MHz, DMSO_d6_) δ_ppm_ 158.6, 157.2, 157.0, 156.6, 153.6, 150.9, 146.5, 137.1, 136.9, 131.8, 130.9, 129.6, 128.9, 128.7, 126.4, 126.2, 123.1, 122.9, 121.7, 121.3, 121.2, 118.9, 118.0, 113.3, 112.6, 112.3, 104.3, 56.1, 21.7. MS (*m/z*) = calcd. for C_34_H_25_N_3_O_4_S: 571.6, found: 571.3.

#### 4-(5-Cyano-6-(1*H*-indol-3-yl)-4-(4-methoxyphenyl)pyridin-2-yl)phenyl 4-methylbenzenesulfonate (2b)

M.P. 170–172 °C, FT-IR (KBr, υ, cm^−1^): 3402, 2900, 2211, 1368, 1153. ^1^H NMR (301 MHz, DMSO_d6_) δ_ppm_ 11.89 (s, 1H, NH), 8.38–8.32 (m, 4H, Aromatic), 7.94 (s, 1H, Aromatic), 7.84–7.80 (m, 4H, Aromatic), 7.58–7.50 (m, 4H, Aromatic), 7.27 (d, *J* = 9 Hz, 3H, Aromatic), 7.19 (d, *J* = 9 Hz, 2H, Aromatic), 3.89 (s, 3H, OCH_3_), 2.45 (s, 3H, CH_3_). ^13^C NMR (76 MHz, DMSO_d6_) δ_ppm_ 161.1, 158.0, 157.2, 155.3, 150.9, 146.5, 137.2, 136.8, 131.8, 131.0, 130.8, 129.7, 129.3, 128.8, 126.5, 123.1, 122.9, 121.7, 121.3, 119.6, 116.9, 114.7, 113.4, 112.6, 102.1, 55.9, 21.7. MS (*m/z*) = calcd. for C_34_H_25_N_3_O_4_S: 571.6, found: 571.3.

#### 4-(4-(4-Chlorophenyl)-5-cyano-6-(1*H*-indol-3-yl)pyridin-2-yl)phenyl 4-methylbenzenesulfonate (2c)

M.P. 188–189 °C, FT-IR (KBr, υ, cm^−1^): 3362, 2922, 2213, 1596, 1433, 1365, 1150. ^1^H NMR (301 MHz, DMSO_d6_) δ_ppm_ 11.90 (s, 1H, NH), 8.39–8.32 (m, 4H, Aromatic), 7.98 (s, 1H, Aromatic), 7.88–7.81 (m, 4H, Aromatic), 7.71 (d, *J* = 9 Hz, 2H, Aromatic), 7.58 (d, *J* = 9 Hz, 1H, Aromatic), 7.51 (d, *J* = 9 Hz, 2H, Aromatic), 7.28–7.24 (m, 4H, Aromatic), 2.44 (s, 3H, CH_3_). MS (*m/z*) = calcd. for C_33_H_22_ClN_3_O_3_S: 576.1, found: 576.2.

#### 4-(5-Cyano-4-(2,4-dichlorophenyl)-6-(*1H*-indol-3-yl)pyridin-2-yl)phenyl 4-methylbenzenesulfonate (2d)

M.P. 205–206 °C, FT-IR (KBr, υ, cm^−1^): 3424, 2221, 1619, 1432, 750. ^1^H NMR (301 MHz, DMSO_d6_) δ_ppm_ 12.00 (s, 1H, NH), 8.39– 8.34 (m, 4H, Aromatic), 8.00 (s, 1H, Aromatic), 7.95 (s, 1H, Aromatic), 7.82 (d, *J* = 9 Hz, 2H, Aromatic), 7.71 (s, 2H, Aromatic), 7.58 (d, *J* = 6 Hz, 1H, Aromatic), 7.51 (d, *J* = 9 Hz, 2H, Aromatic), 7.29–7.26 (m, 4H, Aromatic), 2.44 (s, 3H, CH_3_). ^13^C NMR (76 MHz, DMSO_d6_) δ_ppm_ 162.3, 157.5, 152.7, 151.1, 146.5, 136.9, 135.6, 135.2, 133.2, 132.9, 131.8, 130.8, 129.7, 129.2, 128.8, 128.4, 126.3, 123.2, 121.8, 121.5, 118.2, 117.5, 113.1, 112.7, 103.4, 21.7.

#### 4-(4-Bromophenyl)-2,6-di(1*H*-indol-3-yl) nicotinonitrile (3a)

M.P. 135–137 °C, ^1^H NMR (301 MHz, DMSO_d6_) δ_ppm_ 11.94 (s, 1H, NH), 11.82 (s, 1H, NH), 8.63 (d, *J* = 9 Hz, 1H, Aromatic), 8.53 (d, *J* = 3 Hz, 1H, Aromatic), 8.37 (d, *J* = 9 Hz, 1H, Aromatic), 8.30 (d, *J* = 3 Hz, 1H, Aromatic), 7.90 (s, 1H, Aromatic), 7.85 (d, *J* = 9 Hz, 2H, Aromatic), 7.78 (d, *J* = 9 Hz, 2H, Aromatic), 7.61 (d, *J* = 9 Hz, 1H, Aromatic), 7.54 (d, *J* = 9 Hz, 1H, Aromatic), 7.32–7.20 (m, 3H, Aromatic), 7.10 (t, *J* = 9 Hz, 1H, Aromatic). ^13^C NMR (76 MHz, DMSO_d6_) δ_ppm_ 158.4, 158.1, 152.6, 137.8, 136.9, 132.1, 131.5, 129.9, 128.7, 126.5, 125.8, 123.6, 122.8, 122.6, 121.9, 121.1, 120.8, 119.7, 115.8, 115.3, 114.1, 112.5, 98.8. MS (*m/z*) = calcd. for C_28_H_17_BrN_4_: 489.4, found: 489.1.

#### 4-(4-Chlorophenyl)-2,6-di(1*H*-indol-3-yl) nicotinonitrile (3b)

M.P. 115–120 °C, FT-IR (KBr, υ, cm^−1^): 3391, 2959, 2217, 731. ^1^H NMR (301 MHz, DMSO_d6_) δ_ppm_ 11.92 (s, 1H, NH), 11.81 (s, 1H, NH), 8.61 (d, *J* = 9 Hz, 1H, Aromatic), 8.53 (d, *J* = 9 Hz, 1H, Aromatic), 8.34 (d, *J* = 9 Hz, 1H, Aromatic), 8.28 (d, *J* = 6 Hz, 1H, Aromatic), 7.90 (s, 1H, Aromatic), 7.86 (d, *J* = 9 Hz, 2H, Aromatic), 7.71 (d, *J* = 6 Hz, 2H, Aromatic), 7.59 (d, *J* = 6 Hz, 1H, Aromatic), 7.52 (d, *J* = 9 Hz, 1H, Aromatic), 7.30–7.16 (m, 3H, Aromatic), 7.09 (t, *J* = 9 Hz, 1H, Aromatic). MS (*m/z*) = calcd. for C_28_H_17_ClN_4_: 444.9, found: 444.2.

## Conclusion

In summary, we reported the design, synthesis and characterization of a new heterogeneous catalytic system namely [Fe_3_O_4_@SiO_2_@urea-riched ligand/Ch-Cl]. The revealed results from characterization of this compound such as FT-IR, FESEM, TEM, EDS-Mapping, TGA/DTG and VSM analysis show its successful synthesis. This system has an excellent catalytical potential for synthesis of hybrid pyridines containing sulfonate or indole sections. In this method several starting materials were used for the synthesis of hybrid pyridine rings which yield divers products in mild reaction conditions. Besides, cooperative vinylogous anomeric-based oxidation pathway was suggested as a rational mechanism for the synthesis of hybrid pyridine (Supplementary Information S1).

## Supplementary Information

Supplementary Information.

## Data Availability

All data generated or analyzed during this study are included in this published article [and its supplementary information files].
