# Supplementary material for: Synthesis of new hybrid pyridines catalyzed by Fe3O4@SiO2@urea-riched ligand/Ch-Cl
Source: Sci Rep. 2023 Jun 10;13:9486. doi: 10.1038/s41598-023-35849-3 (PMC10257680; doi:10.1038/s41598-023-35849-3)

**Synthesis of new hybrid pyridines catalyzed by Fe_3_O_4_@SiO_2_@urea-riched ligand/Ch-Cl** Narges Zarei, Mohammad Ali Zolfigol,* Morteza Torabi, Meysam Yarie*

Department of Organic Chemistry, Faculty of Chemistry, Bu-Ali Sina University, Hamedan, Iran

zolfi@basu.ac.ir; [mzolfigol@yahoo.com](mailto:mzolfigol@yahoo.com); myarie.5266@gmail.com.

1: FT-IR spectrum of Fe_3_O_4_@SiO_2_@urea-riched ligand/Ch-Cl.

2: FT-IR spectrum of recycled Fe_3_O_4_@SiO_2_@urea-riched ligand/Ch-Cl.

3: FT-IR spectrum of 4-(6-amino-5-cyano-4-phenylpyridin-2-yl)phenyl 4-methylbenzenesulfonate. (1a)

4: ^1^H NMR spectrum of 4-(6-amino-5-cyano-4-phenylpyridin-2-yl)phenyl 4-methylbenzenesulfonate. (1a)

5: ^13^C NMR spectrum of 4-(6-amino-5-cyano-4-phenylpyridin-2-yl)phenyl 4-methylbenzenesulfonate. (1a)

6: Mass spectrum of 4-(6-amino-5-cyano-4-phenylpyridin-2-yl)phenyl 4-methylbenzenesulfonate. (1a)

7: FT-IR spectrum of 4-(6-amino-5-cyano-4-(*p*-tolyl)pyridin-2-yl)phenyl 4-methylbenzenesulfonate. (1b)

8: ^1^H NMR spectrum of 4-(6-amino-5-cyano-4-(*p*-tolyl)pyridin-2-yl)phenyl 4-methylbenzenesulfonate. (1b)

9: ^13^C NMR spectrum of 4-(6-amino-5-cyano-4-(*p*-tolyl)pyridin-2-yl)phenyl 4-methylbenzenesulfonate. (1b)

10: Mass spectrum of 4-(6-amino-5-cyano-4-(*p*-tolyl)pyridin-2-yl)phenyl 4-methylbenzenesulfonate. (1b)

11: FT-IR spectrum of 4-(6-amino-5-cyano-4-(4-methoxyphenyl)pyridin-2-yl)phenyl 4-methylbenzenesulfonate. (1c)

12: ^1^H NMR spectrum of 4-(6-amino-5-cyano-4-(4-methoxyphenyl)pyridin-2-yl)phenyl 4-methylbenzenesulfonate. (1c)

13: ^13^C NMR spectrum of 4-(6-amino-5-cyano-4-(4-methoxyphenyl)pyridin-2-yl)phenyl 4-methylbenzenesulfonate. (1c)

14: Mass spectrum of 4-(6-amino-5-cyano-4-(4-methoxyphenyl)pyridin-2-yl)phenyl 4-methylbenzenesulfonate. (1c)

15: FT-IR spectrum of 4-(4-([1,1'-biphenyl]-4-yl)-6-amino-5-cyanopyridin-2-yl)phenyl 4-methylbenzenesulfonate. (1d)

16: ^1^H NMR spectrum of 4-(4-([1,1'-biphenyl]-4-yl)-6-amino-5-cyanopyridin-2-yl)phenyl 4-methylbenzenesulfonate. (1d)

17: ^13^C NMR spectrum of 4-(4-([1,1'-biphenyl]-4-yl)-6-amino-5-cyanopyridin-2-yl)phenyl 4-methylbenzenesulfonate. (1d)

18: Mass spectrum of 4-(4-([1,1'-biphenyl]-4-yl)-6-amino-5-cyanopyridin-2-yl)phenyl 4-methylbenzenesulfonate. (1d)

19: FT-IR spectrum of 4-(6'-amino-5'-cyano-[3,4'-bipyridin]-2'-yl)phenyl 4-methylbenzenesulfonate. (1e)

20: ^1^H NMR spectrum of 4-(6'-amino-5'-cyano-[3,4'-bipyridin]-2'-yl)phenyl 4-methylbenzenesulfonate. (1e)

21: Mass spectrum of 4-(6'-amino-5'-cyano-[3,4'-bipyridin]-2'-yl)phenyl 4-methylbenzenesulfonate. (1e)

22: FT-IR spectrum of 4-(6'-amino-5'-cyano-[3,4'-bipyridin]-2'-yl)phenyl benzenesulfonate. (1f)

23: ^1^H NMR spectrum of 4-(6'-amino-5'-cyano-[3,4'-bipyridin]-2'-yl)phenyl benzenesulfonate. (1f)

24: ^13^C NMR spectrum of 4-(6'-amino-5'-cyano-[3,4'-bipyridin]-2'-yl)phenyl benzenesulfonate. (1f)

25: Mass spectrum of 4-(6'-amino-5'-cyano-[3,4'-bipyridin]-2'-yl)phenyl benzenesulfonate. (1f)

26: FT-IR spectrum of 4-(6-amino-5-cyano-[4,4'-bipyridin]-2-yl)phenylbenzenesulfonate. (1g)

27: ^1^H NMR spectrum of 4-(6-amino-5-cyano-[4,4'-bipyridin]-2-yl)phenylbenzenesulfonate. (1g)

28: ^13^C NMR spectrum of 4-(6-amino-5-cyano-[4,4'-bipyridin]-2-yl)phenylbenzenesulfonate. (1g)

29: Mass spectrum of 4-(6-amino-5-cyano-[4,4'-bipyridin]-2-yl)phenylbenzenesulfonate. (1g)

30: FT-IR spectrum of 4-(6-amino-4-(2-chlorophenyl)-5-cyanopyridin-2-yl)phenyl 4-methylbenzenesulfonate. (1h)

31: ^1^H NMR spectrum of 4-(6-amino-4-(2-chlorophenyl)-5-cyanopyridin-2-yl)phenyl 4-methylbenzenesulfonate. (1h)

32: ^13^C NMR spectrum of 4-(6-amino-4-(2-chlorophenyl)-5-cyanopyridin-2-yl)phenyl 4-methylbenzenesulfonate. (1h)

33: Mass spectrum of 4-(6-amino-4-(2-chlorophenyl)-5-cyanopyridin-2-yl)phenyl 4-methylbenzenesulfonate. (1h)

34: FT-IR spectrum of 4-(6-amino-5-cyano-4-(2,4-dichlorophenyl)pyridin-2-yl)phenyl 4-methylbenzenesulfonate. (1i)

35: ^1^H NMR spectrum of 4-(6-amino-5-cyano-4-(2,4-dichlorophenyl)pyridin-2-yl)phenyl 4-methylbenzenesulfonate. (1i)

36: ^13^C NMR spectrum of 4-(6-amino-5-cyano-4-(2,4-dichlorophenyl)pyridin-2-yl)phenyl 4-methylbenzenesulfonate. (1i)

37: Mass spectrum of 4-(6-amino-5-cyano-4-(2,4-dichlorophenyl)pyridin-2-yl)phenyl 4-methylbenzenesulfonate. (1i)

38: FT-IR spectrum of 4-(6-amino-5-cyano-4-(3-fluorophenyl)pyridin-2-yl)phenyl 4-methylbenzenesulfonate. (1j)

39: ^1^H NMR spectrum of 4-(6-amino-5-cyano-4-(3-fluorophenyl)pyridin-2-yl)phenyl 4-methylbenzenesulfonate. (1j)

40: ^13^C NMR spectrum of 4-(6-amino-5-cyano-4-(3-fluorophenyl)pyridin-2-yl)phenyl 4-methylbenzenesulfonate. (1j)

41: Mass spectrum of 4-(6-amino-5-cyano-4-(3-fluorophenyl)pyridin-2-yl)phenyl 4-methylbenzenesulfonate. (1j)

42: FT-IR spectrum of 4-(6-amino-4-(4-bromophenyl)-5-cyanopyridin-2-yl)phenyl 4-methylbenzenesulfonate. (1k)

43: ^1^H NMR spectrum of 4-(6-amino-4-(4-bromophenyl)-5-cyanopyridin-2-yl)phenyl 4-methylbenzenesulfonate. (1k)

44: ^13^C NMR spectrum of 4-(6-amino-4-(4-bromophenyl)-5-cyanopyridin-2-yl)phenyl 4-methylbenzenesulfonate. (1k)

45: Mass spectrum of 4-(6-amino-4-(4-bromophenyl)-5-cyanopyridin-2-yl)phenyl 4-methylbenzenesulfonate. (1k)

46: FT-IR spectrum of 4-(5-cyano-6-(1*H*-indol-3-yl)-4-(2-methoxyphenyl)pyridin-2-yl)phenyl 4-methylbenzenesulfonate. (2a)

47: ^1^H NMR spectrum of 4-(5-cyano-6-(1*H*-indol-3-yl)-4-(2-methoxyphenyl)pyridin-2-yl)phenyl 4-methylbenzenesulfonate. (2a)

48: ^13^C NMR spectrum of 4-(5-cyano-6-(1*H*-indol-3-yl)-4-(2-methoxyphenyl)pyridin-2-yl)phenyl 4-methylbenzenesulfonate. (2a)

49: Mass spectrum of 4-(5-cyano-6-(1*H*-indol-3-yl)-4-(2-methoxyphenyl)pyridin-2-yl)phenyl 4-methylbenzenesulfonate. (2a)

50: FT-IR spectrum of 4-(5-cyano-6-(1*H*-indol-3-yl)-4-(4-methoxyphenyl)pyridin-2-yl)phenyl 4-methylbenzenesulfonate. (2b)

51: ^1^H NMR spectrum of 4-(5-cyano-6-(1*H*-indol-3-yl)-4-(4-methoxyphenyl)pyridin-2-yl)phenyl 4-methylbenzenesulfonate. (2b)

52: ^13^C NMR spectrum of 4-(5-cyano-6-(1*H*-indol-3-yl)-4-(4-methoxyphenyl)pyridin-2-yl)phenyl 4-methylbenzenesulfonate. (2b)

53: Mass spectrum of 4-(5-cyano-6-(1*H*-indol-3-yl)-4-(4-methoxyphenyl)pyridin-2-yl)phenyl 4-methylbenzenesulfonate. (2b)

54: FT-IR spectrum of 4-(4-(4-chlorophenyl)-5-cyano-6-(1*H*-indol-3-yl)pyridin-2-yl)phenyl 4-methylbenzenesulfonate. (2c)

55: ^1^H NMR spectrum of 4-(4-(4-chlorophenyl)-5-cyano-6-(1*H*-indol-3-yl)pyridin-2-yl)phenyl 4-methylbenzenesulfonate. (2c)

56: Mass spectrum of 4-(4-(4-chlorophenyl)-5-cyano-6-(1*H*-indol-3-yl)pyridin-2-yl)phenyl 4-methylbenzenesulfonate. (2c)

57: FT-IR spectrum of 4-(5-cyano-4-(2,4-dichlorophenyl)-6-(1*H*-indol-3-yl)pyridin-2-yl)phenyl 4-methylbenzenesulfonate. (2d)

58: ^1^H NMR spectrum of 4-(5-cyano-4-(2,4-dichlorophenyl)-6-(1*H*-indol-3-yl)pyridin-2-yl)phenyl 4-methylbenzenesulfonate. (2d)

59: ^13^C NMR spectrum of 4-(5-cyano-4-(2,4-dichlorophenyl)-6-(1*H*-indol-3-yl)pyridin-2-yl)phenyl 4-methylbenzenesulfonate. (2d)

60: ^1^H NMR spectrum of 4-(4-bromophenyl)-2,6-di(1*H*-indol-3-yl) nicotinonitrile. (3a)

61: ^13^C NMR spectrum of 4-(4-bromophenyl)-2,6-di(1*H*-indol-3-yl) nicotinonitrile. (3a)

62: Mass spectrum of 4-(4-bromophenyl)-2,6-di(1*H*-indol-3-yl) nicotinonitrile. (3a)

63: FT-IR spectrum of 4-(4-chlorophenyl)-2,6-di(1*H*-indol-3-yl) nicotinonitrile. (3b)

64: ^1^H NMR spectrum of 4-(4-chlorophenyl)-2,6-di(1*H*-indol-3-yl) nicotinonitrile. (3b)

65: Mass spectrum of 4-(4-chlorophenyl)-2,6-di(1*H*-indol-3-yl) nicotinonitrile. (3b)

66: FT-IR spectrum of Ch-Cl/urea

67: FT-IR spectrum of Ch-Cl/thiourea

68: FT-IR spectrum of Ch-Cl/acetamide

69: FT-IR spectrum of Ch-Cl/benzoic acid

70: FT-IR spectrum of Ch-Cl/ascorbic acid

1: FT-IR spectrum of Fe_3_O_4_@SiO_2_@urea-riched ligand/Ch-Cl.


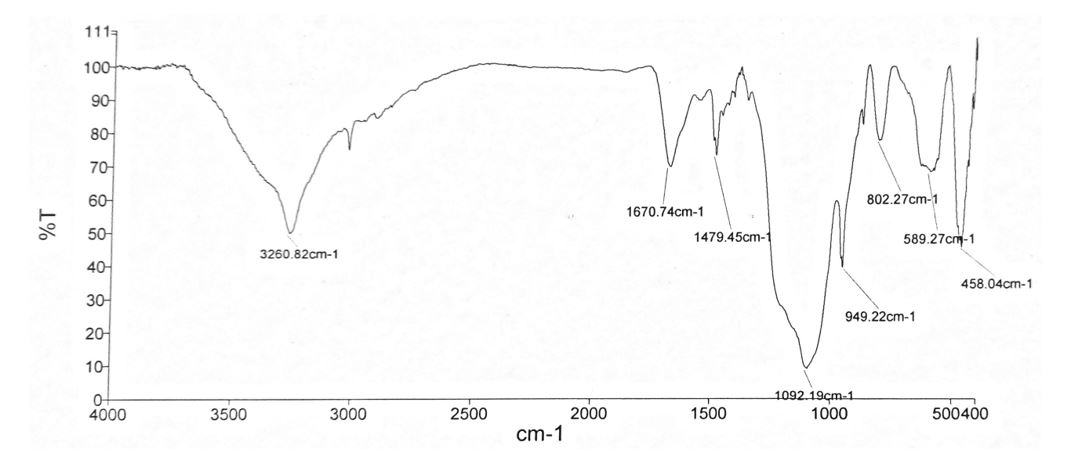


2: FT-IR spectrum of recycled Fe_3_O_4_@SiO_2_@urea-riched ligand/Ch-Cl.


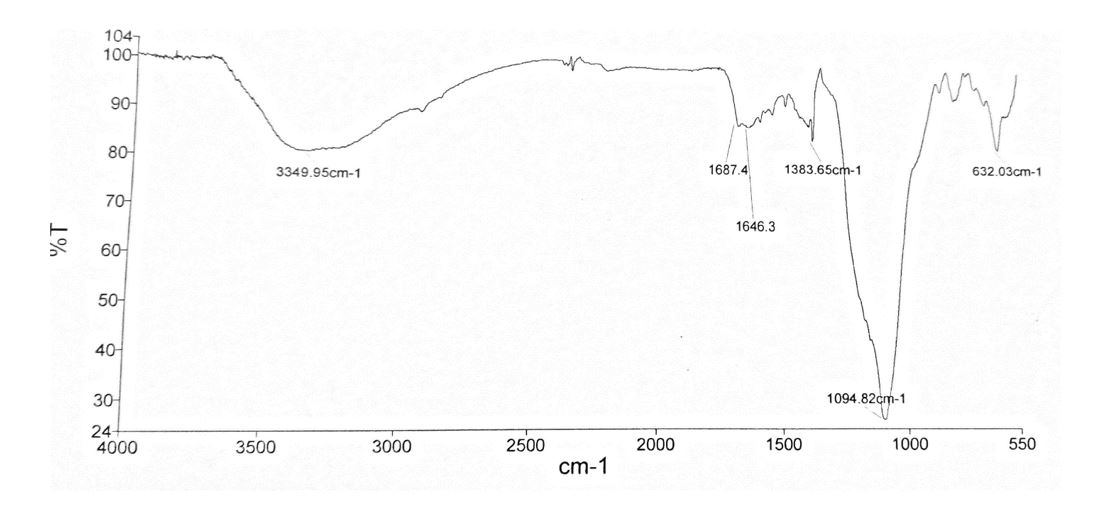


3: FT-IR spectrum of 4-(6-amino-5-cyano-4-phenylpyridin-2-yl)phenyl 4-methylbenzenesulfonate. (1a)


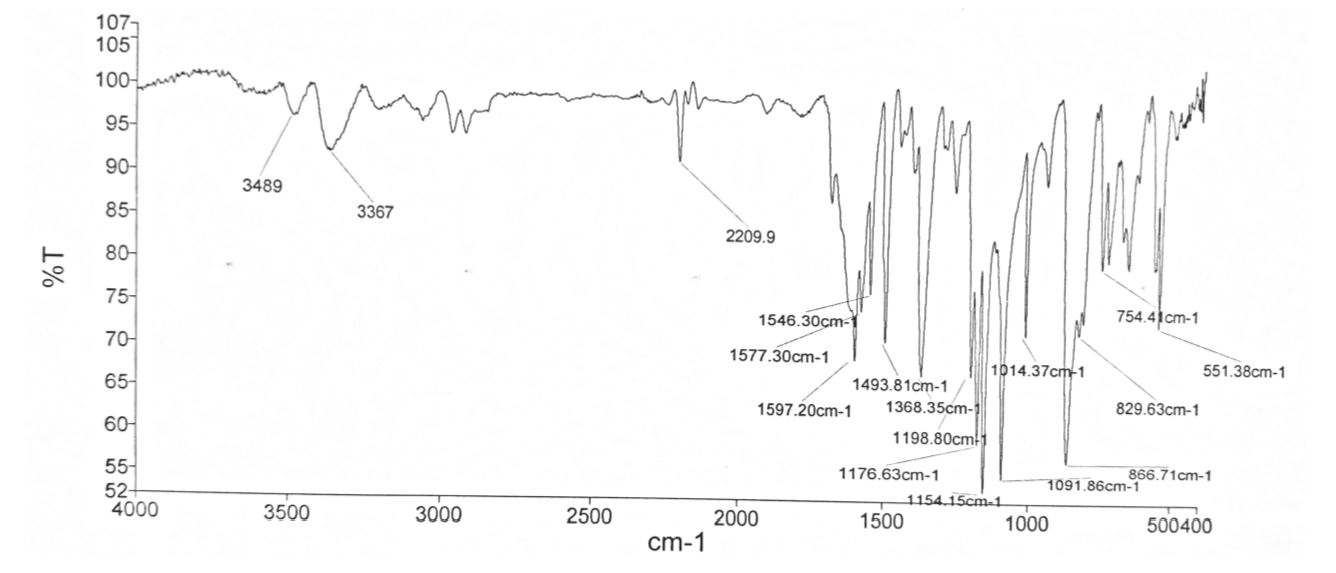
4: ^1^H NMR spectrum of 4-(6-amino-5-cyano-4-phenylpyridin-2-yl)phenyl 4-methylbenzenesulfonate. (1a)


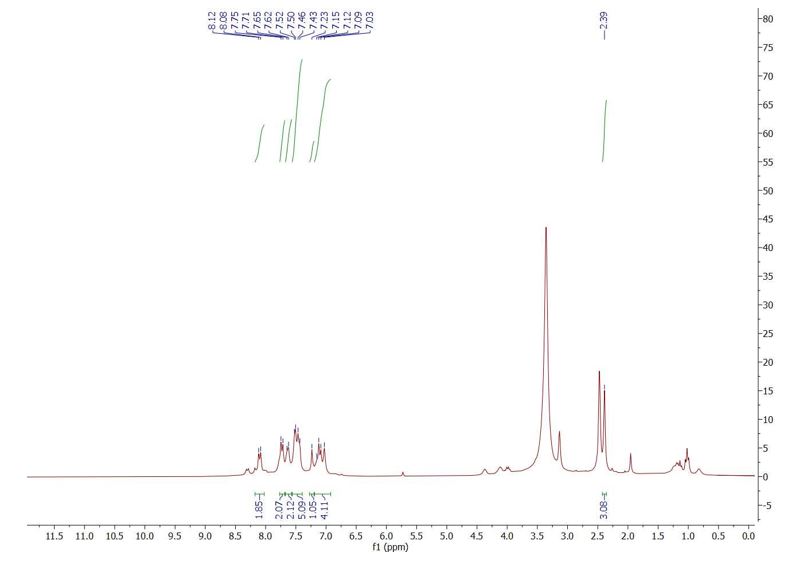


5: ^13^C NMR spectrum of 4-(6-amino-5-cyano-4-phenylpyridin-2-yl)phenyl 4-methylbenzenesulfonate. (1a)


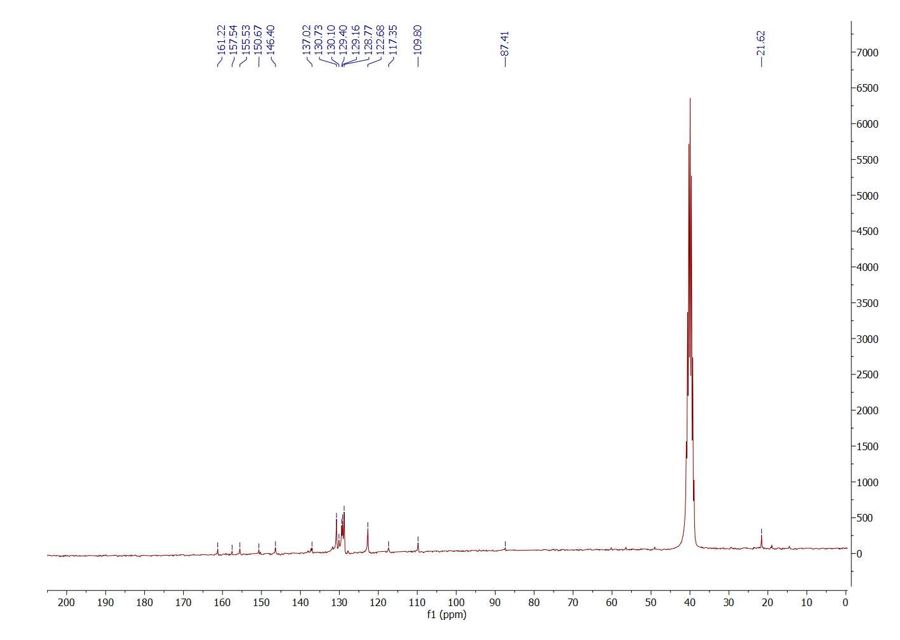


6: Mass spectrum of 4-(6-amino-5-cyano-4-phenylpyridin-2-yl)phenyl 4-methylbenzenesulfonate. (1a)


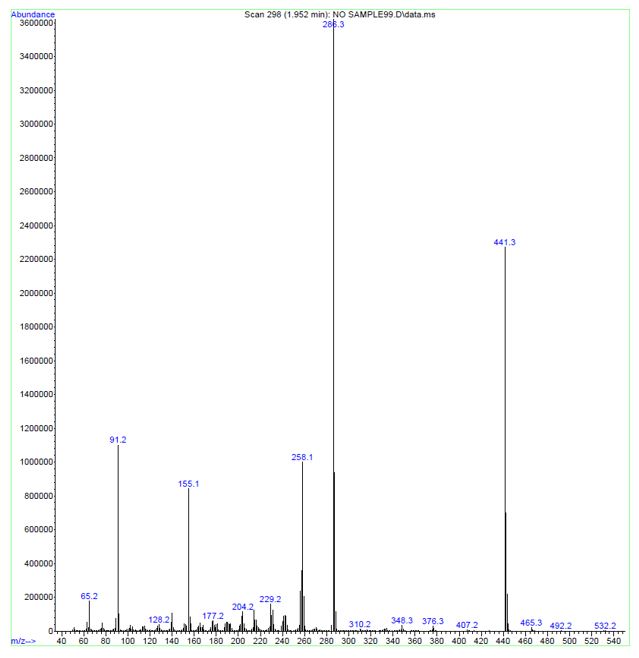


7: FT-IR spectrum of 4-(6-amino-5-cyano-4-(*p*-tolyl)pyridin-2-yl)phenyl 4-methylbenzenesulfonate. (1b)


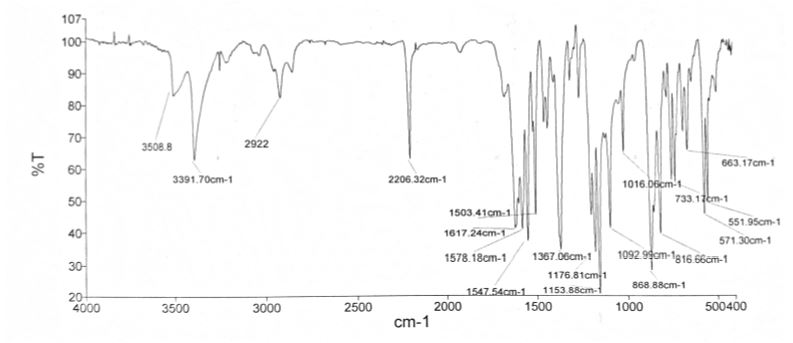
8: ^1^H NMR spectrum of 4-(6-amino-5-cyano-4-(*p*-tolyl)pyridin-2-yl)phenyl 4-methylbenzenesulfonate. (1b)


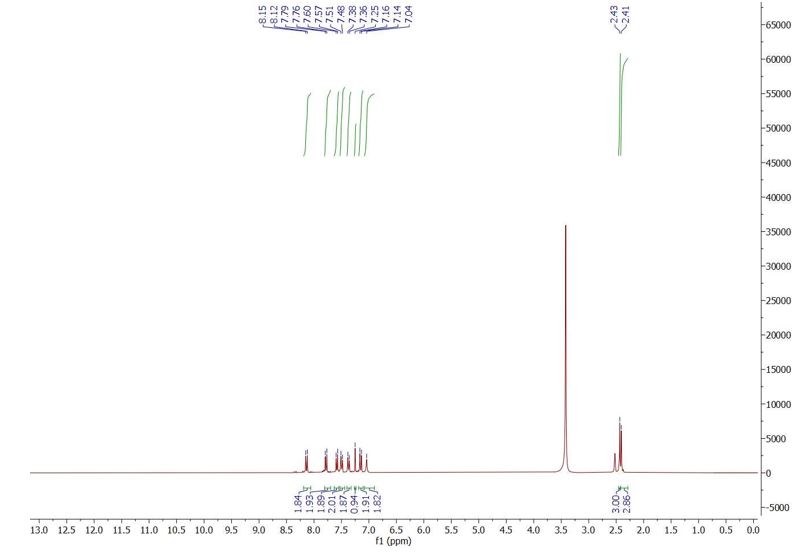


9: ^13^C NMR spectrum of 4-(6-amino-5-cyano-4-(*p*-tolyl)pyridin-2-yl)phenyl 4-methylbenzenesulfonate. (1b)


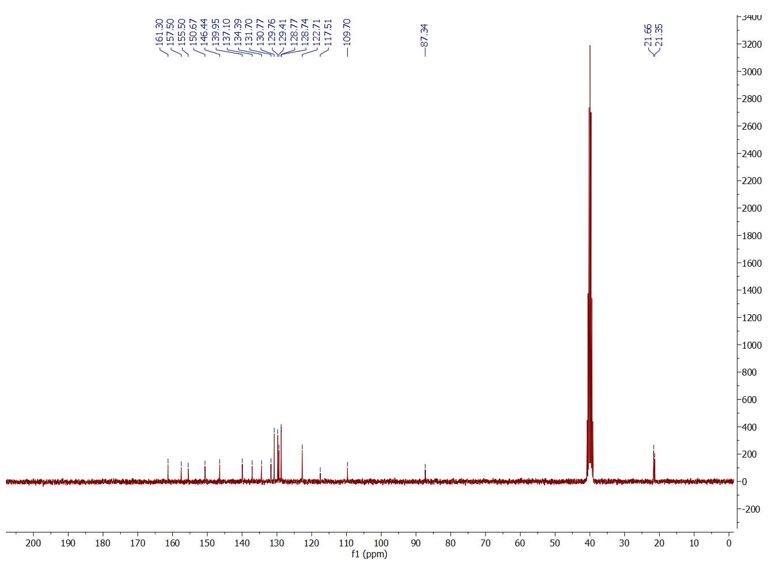


10: Mass spectrum of 4-(6-amino-5-cyano-4-(*p*-tolyl)pyridin-2-yl)phenyl 4-methylbenzenesulfonate. (1b)


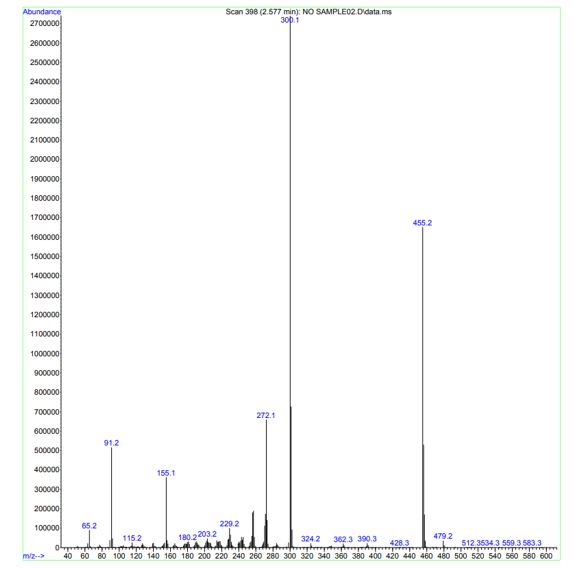


11: FT-IR spectrum of 4-(6-amino-5-cyano-4-(4-methoxyphenyl)pyridin-2-yl)phenyl 4-methylbenzenesulfonate. (1c)


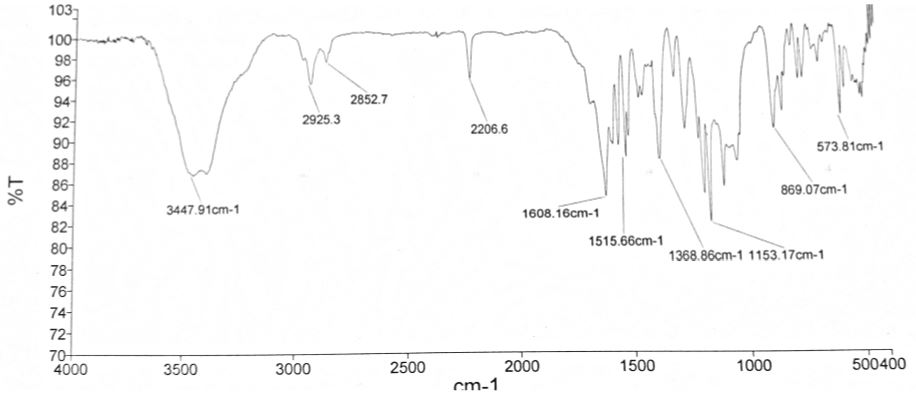


12: ^1^H NMR spectrum of 4-(6-amino-5-cyano-4-(4-methoxyphenyl)pyridin-2-yl)phenyl 4-methylbenzenesulfonate. (1c)


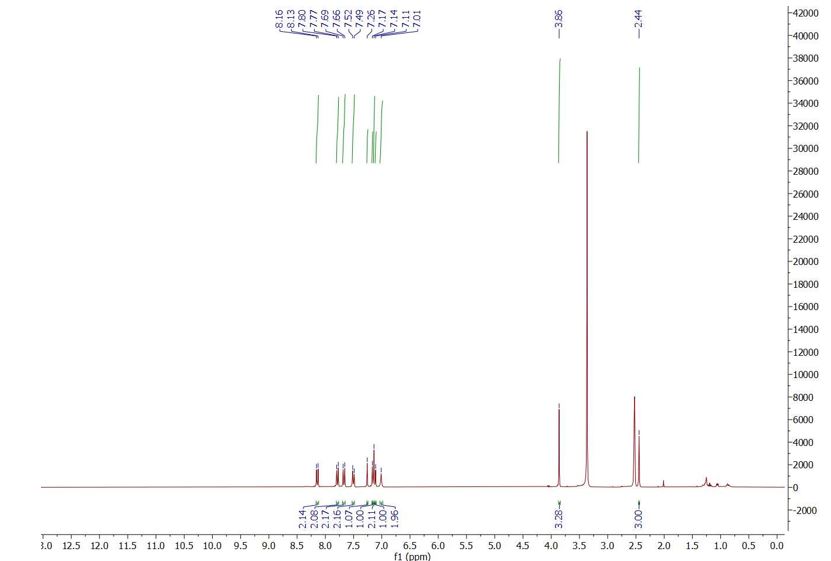


13: ^13^C NMR spectrum of 4-(6-amino-5-cyano-4-(4-methoxyphenyl)pyridin-2-yl)phenyl 4-methylbenzenesulfonate. (1c)


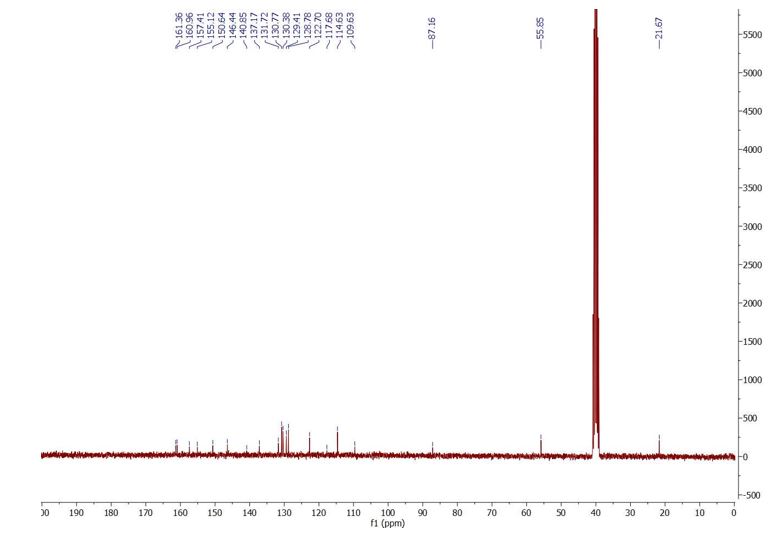


14: Mass spectrum of 4-(6-amino-5-cyano-4-(4-methoxyphenyl)pyridin-2-yl)phenyl 4-methylbenzenesulfonate. (1c)


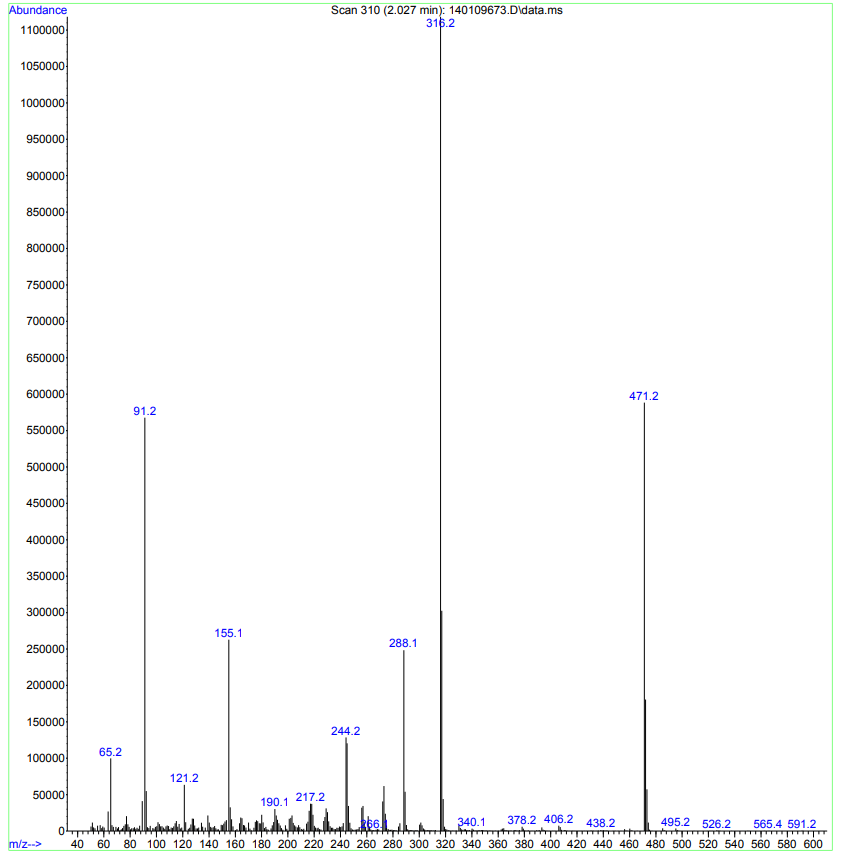


15: FT-IR spectrum of 4-(4-([1,1'-biphenyl]-4-yl)-6-amino-5-cyanopyridin-2-yl)phenyl 4-methylbenzenesulfonate. (1d)


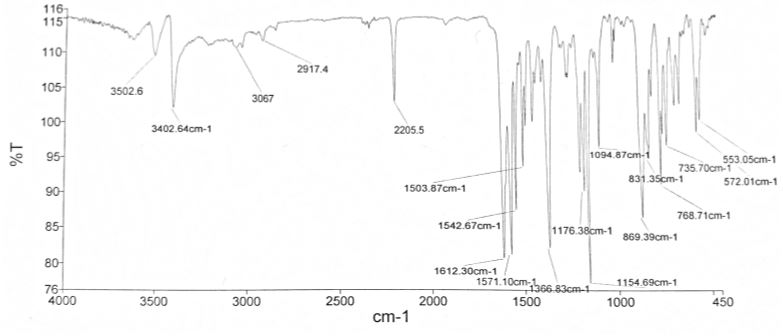


16: ^1^H NMR spectrum of 4-(4-([1,1'-biphenyl]-4-yl)-6-amino-5-cyanopyridin-2-yl)phenyl 4-methylbenzenesulfonate. (1d)


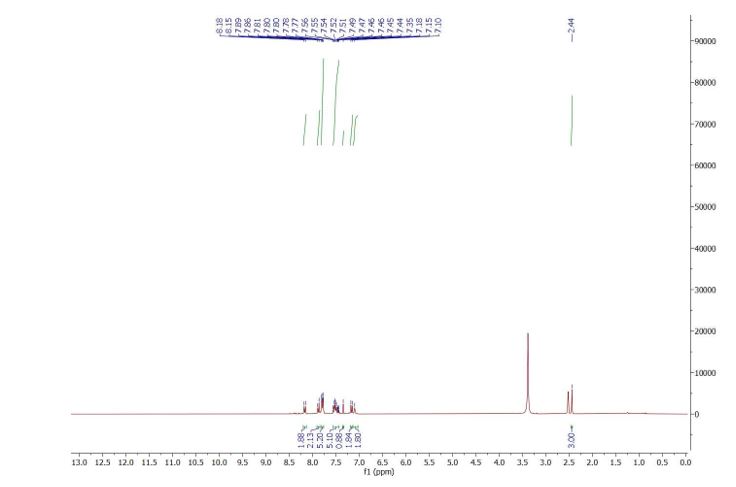


17: ^13^C NMR spectrum of 4-(4-([1,1'-biphenyl]-4-yl)-6-amino-5-cyanopyridin-2-yl)phenyl 4-methylbenzenesulfonate. (1d)


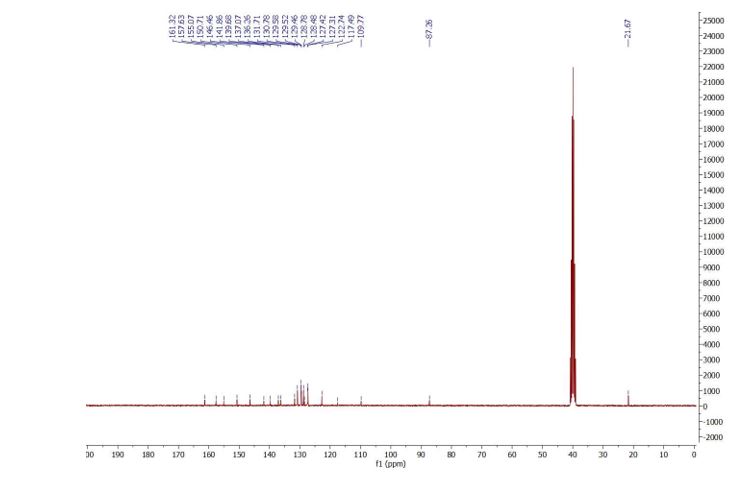


18: Mass spectrum of 4-(4-([1,1'-biphenyl]-4-yl)-6-amino-5-cyanopyridin-2-yl)phenyl 4-methylbenzenesulfonate. (1d)


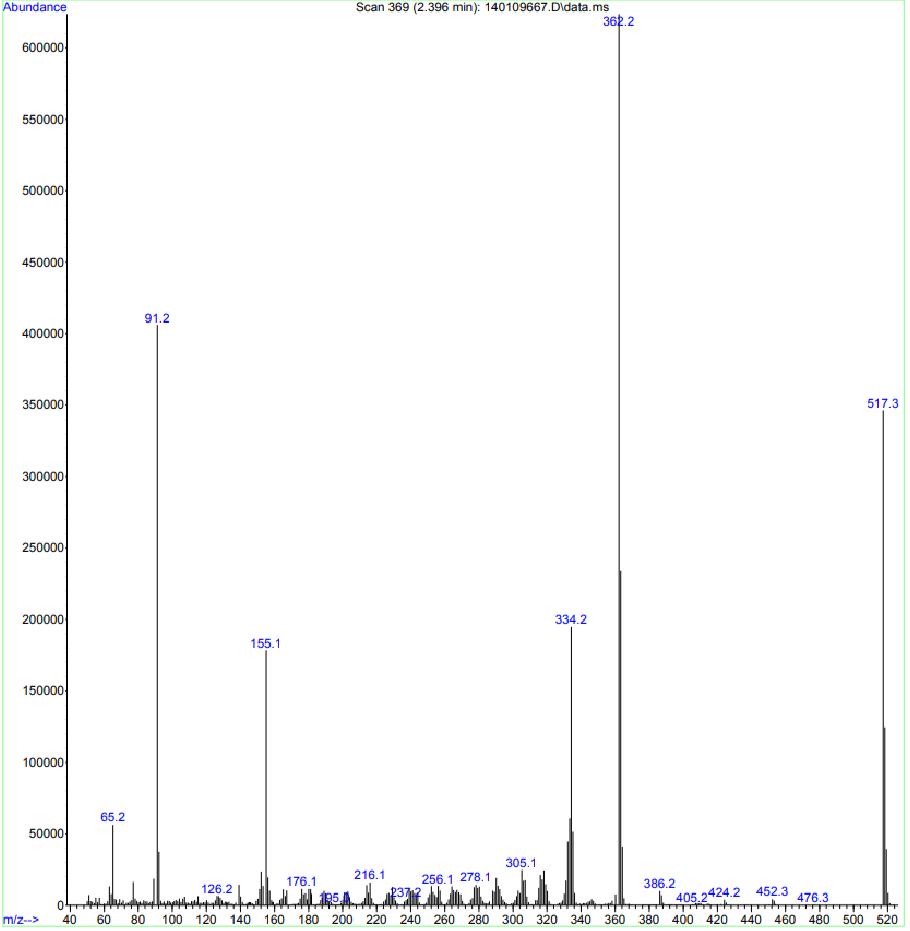


19: FT-IR spectrum of 4-(6'-amino-5'-cyano-[3,4'-bipyridin]-2'-yl)phenyl 4-methylbenzenesulfonate. (1e)


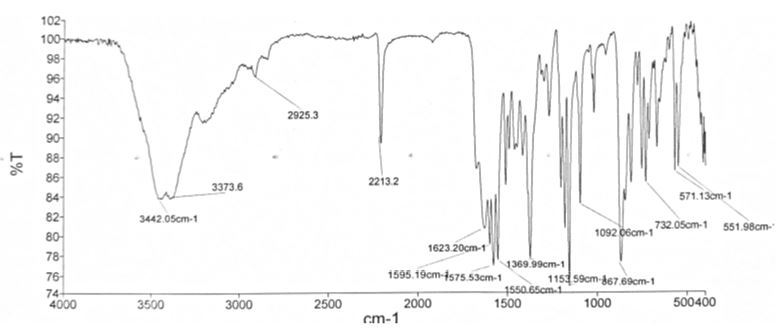


20: ^1^H NMR spectrum of 4-(6'-amino-5'-cyano-[3,4'-bipyridin]-2'-yl)phenyl 4-methylbenzenesulfonate. (1e)


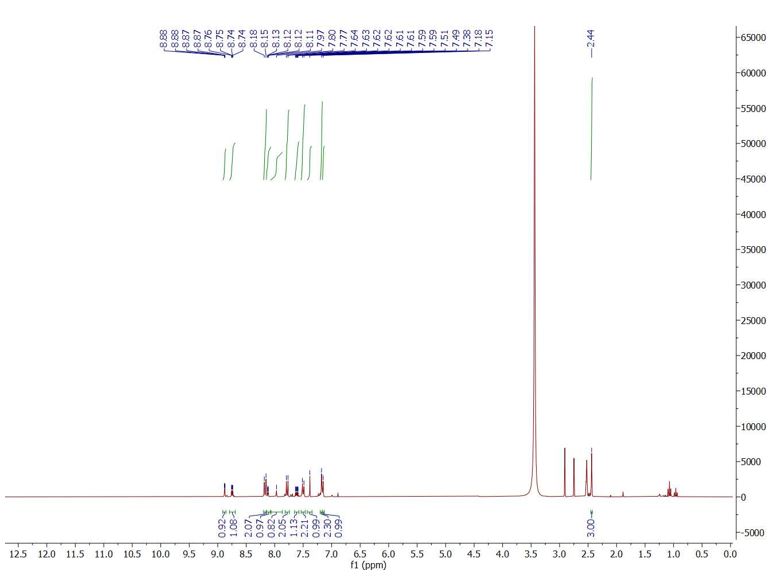


21: Mass spectrum of 4-(6'-amino-5'-cyano-[3,4'-bipyridin]-2'-yl)phenyl 4-methylbenzenesulfonate. (1e)


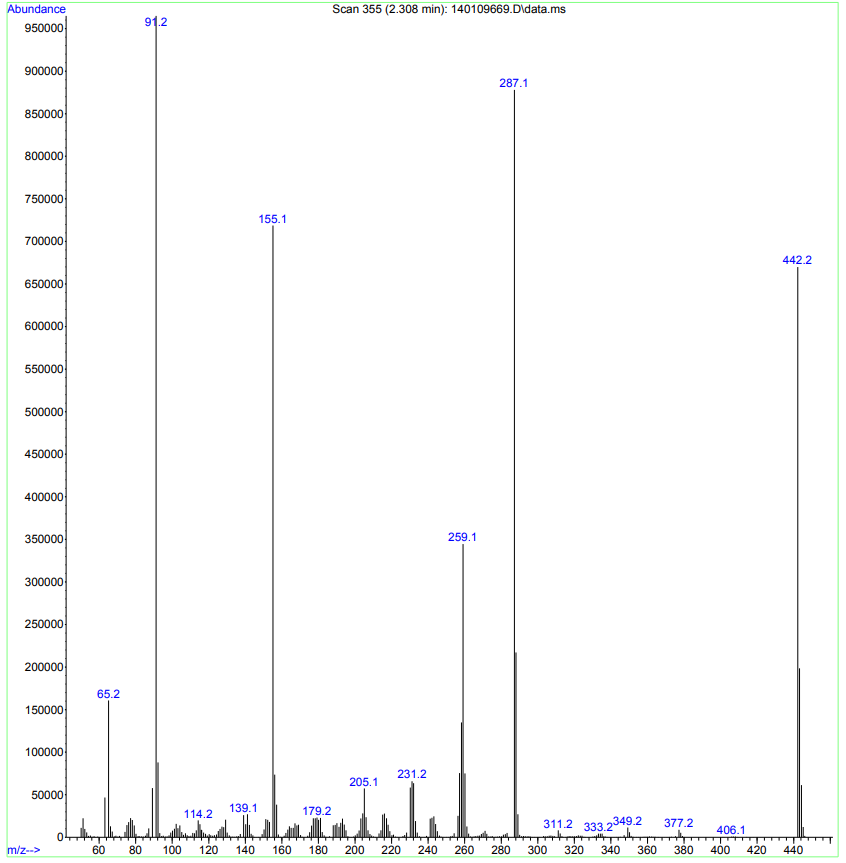


22: FT-IR spectrum of 4-(6'-amino-5'-cyano-[3,4'-bipyridin]-2'-yl)phenyl benzenesulfonate. (1f)


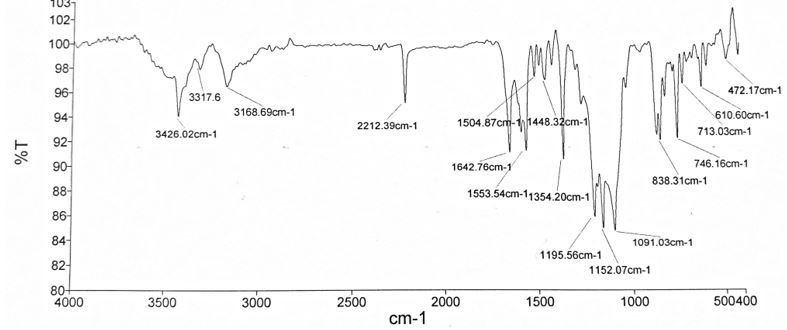


23: ^1^H NMR spectrum of 4-(6'-amino-5'-cyano-[3,4'-bipyridin]-2'-yl)phenyl benzenesulfonate. (1f)


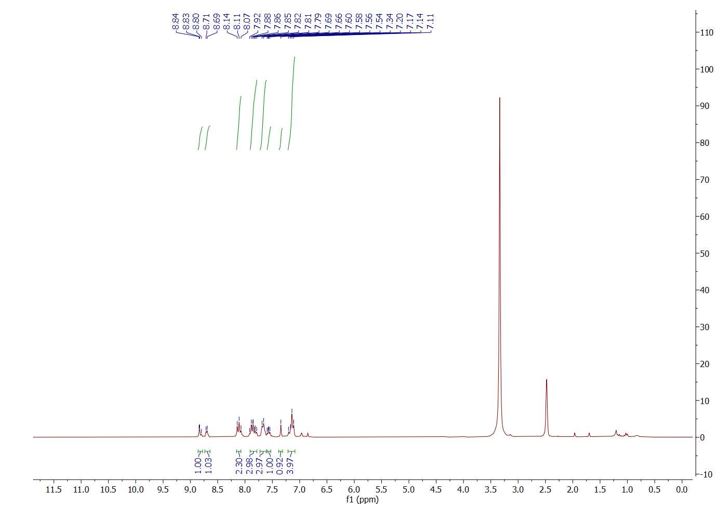


24: ^13^C NMR spectrum of 4-(6'-amino-5'-cyano-[3,4'-bipyridin]-2'-yl)phenyl benzenesulfonate. (1f)


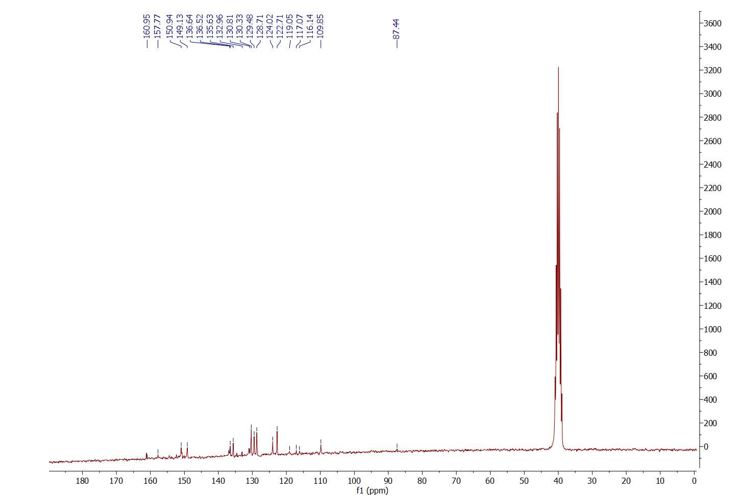


25: Mass spectrum of 4-(6'-amino-5'-cyano-[3,4'-bipyridin]-2'-yl)phenyl benzenesulfonate. (1f)


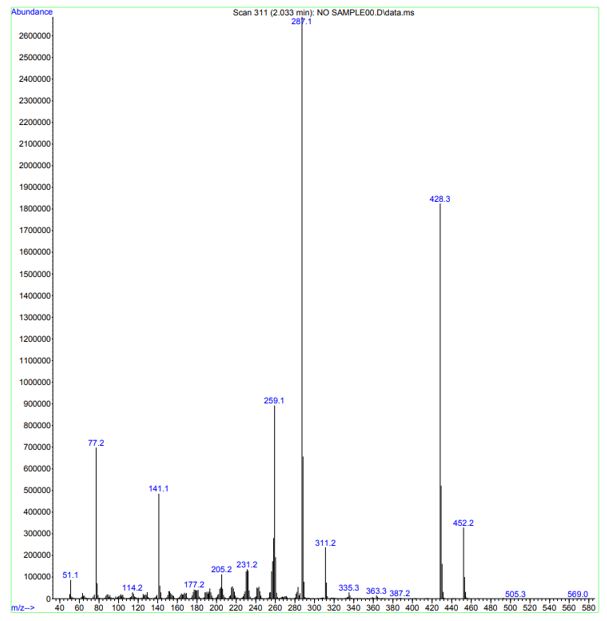


26: FT-IR spectrum of 4-(6-amino-5-cyano-[4,4'-bipyridin]-2-yl)phenylbenzenesulfonate. (1g)


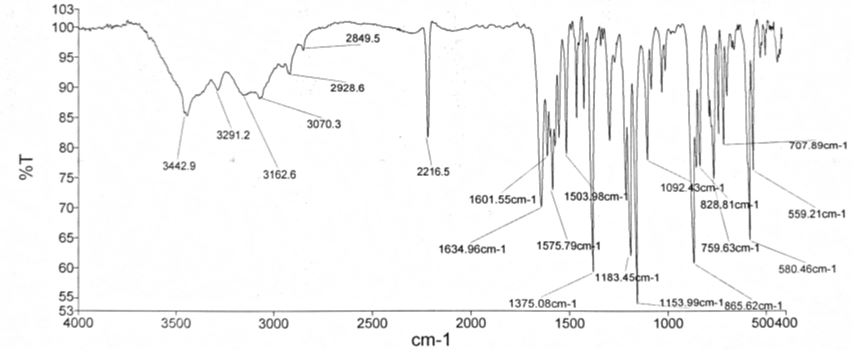


27: ^1^H NMR spectrum of 4-(6-amino-5-cyano-[4,4'-bipyridin]-2-yl)phenylbenzenesulfonate. (1g)


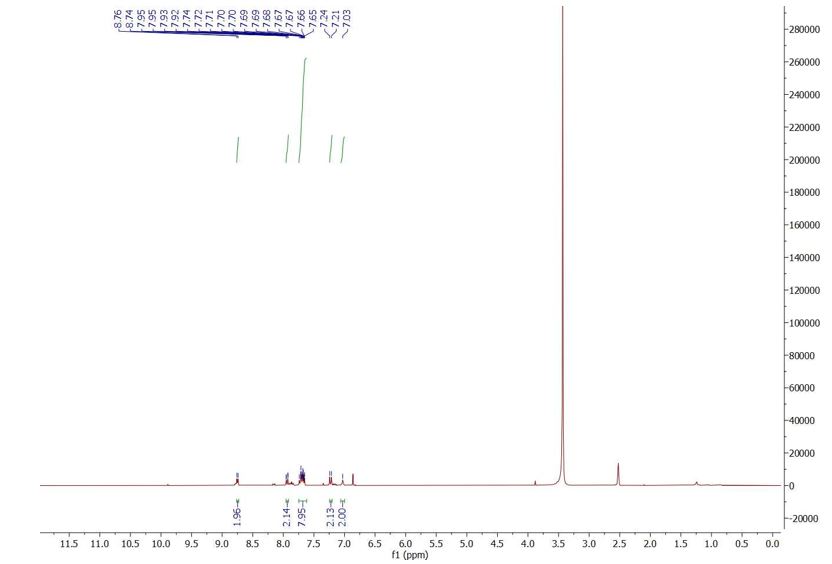


28: ^13^C NMR spectrum of 4-(6-amino-5-cyano-[4,4'-bipyridin]-2-yl)phenylbenzenesulfonate. (1g)


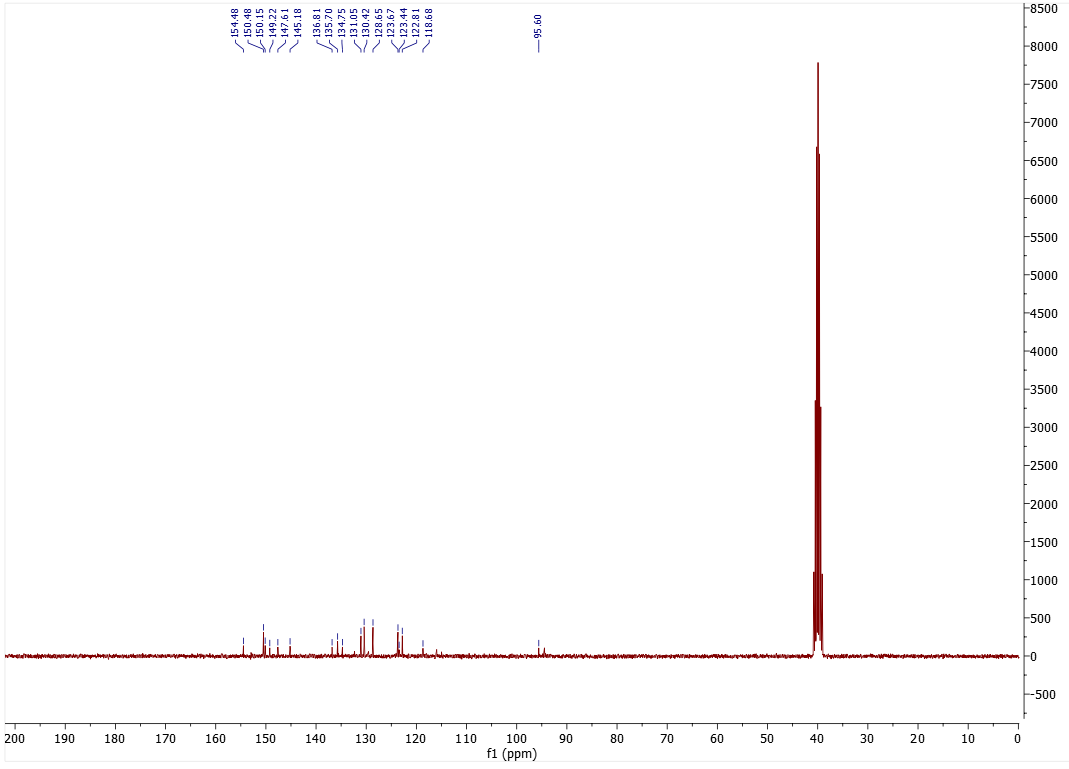


29: Mass spectrum of 4-(6-amino-5-cyano-[4,4'-bipyridin]-2-yl)phenylbenzenesulfonate. (1g)


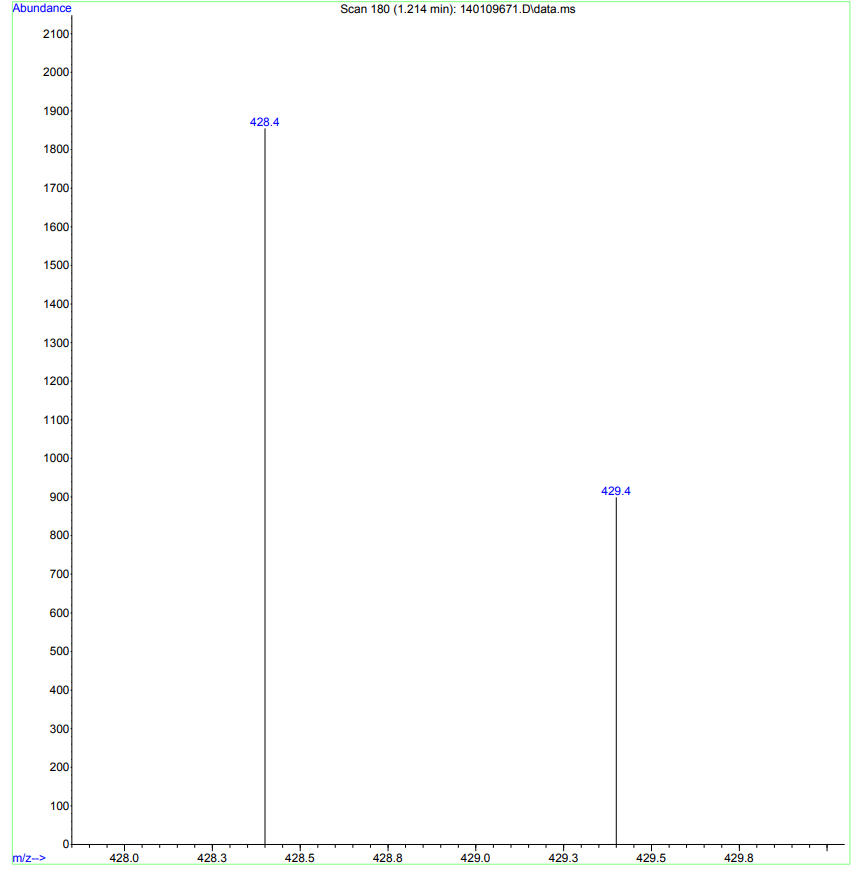


30: FT-IR spectrum of 4-(6-amino-4-(2-chlorophenyl)-5-cyanopyridin-2-yl)phenyl 4-methylbenzenesulfonate. (1h)


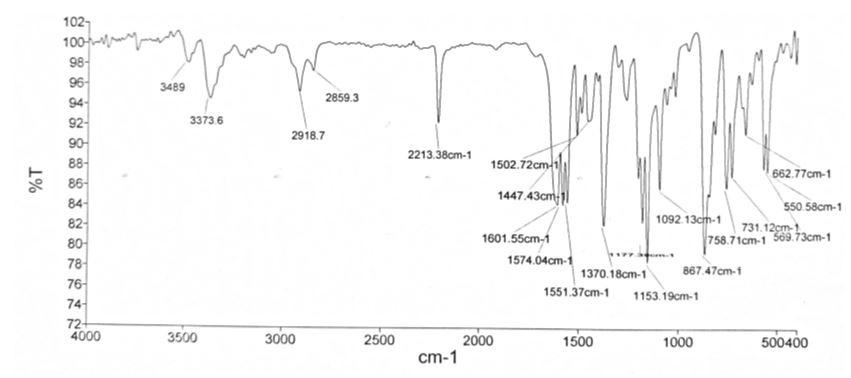

31: ^1^H NMR spectrum of 4-(6-amino-4-(2-chlorophenyl)-5-cyanopyridin-2-yl)phenyl 4-methylbenzenesulfonate. (1h)


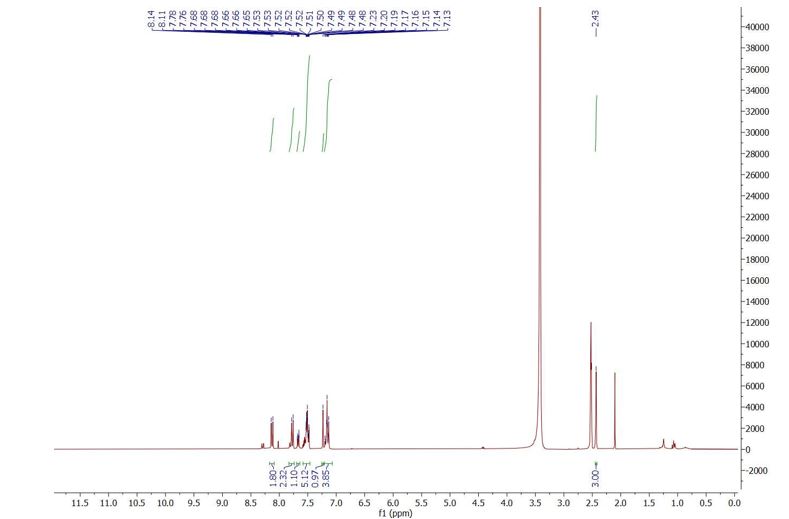


32: ^13^C NMR spectrum of 4-(6-amino-4-(2-chlorophenyl)-5-cyanopyridin-2-yl)phenyl 4-methylbenzenesulfonate. (1h)


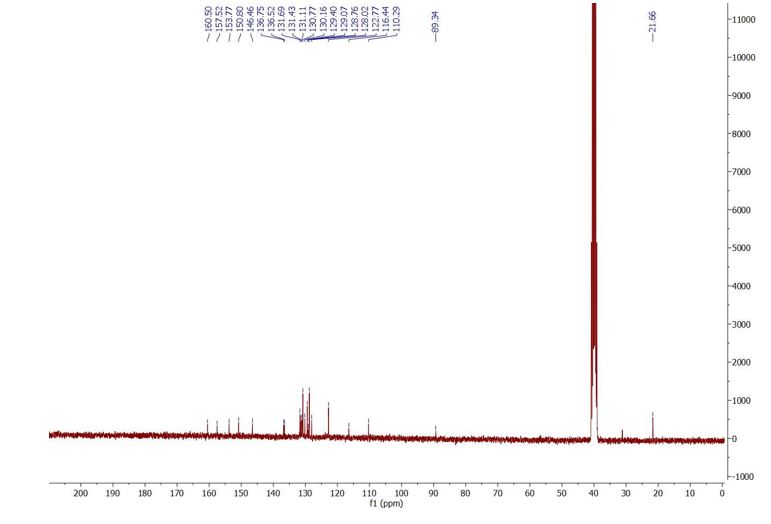


33: Mass spectrum of 4-(6-amino-4-(2-chlorophenyl)-5-cyanopyridin-2-yl)phenyl 4-methylbenzenesulfonate. (1h)


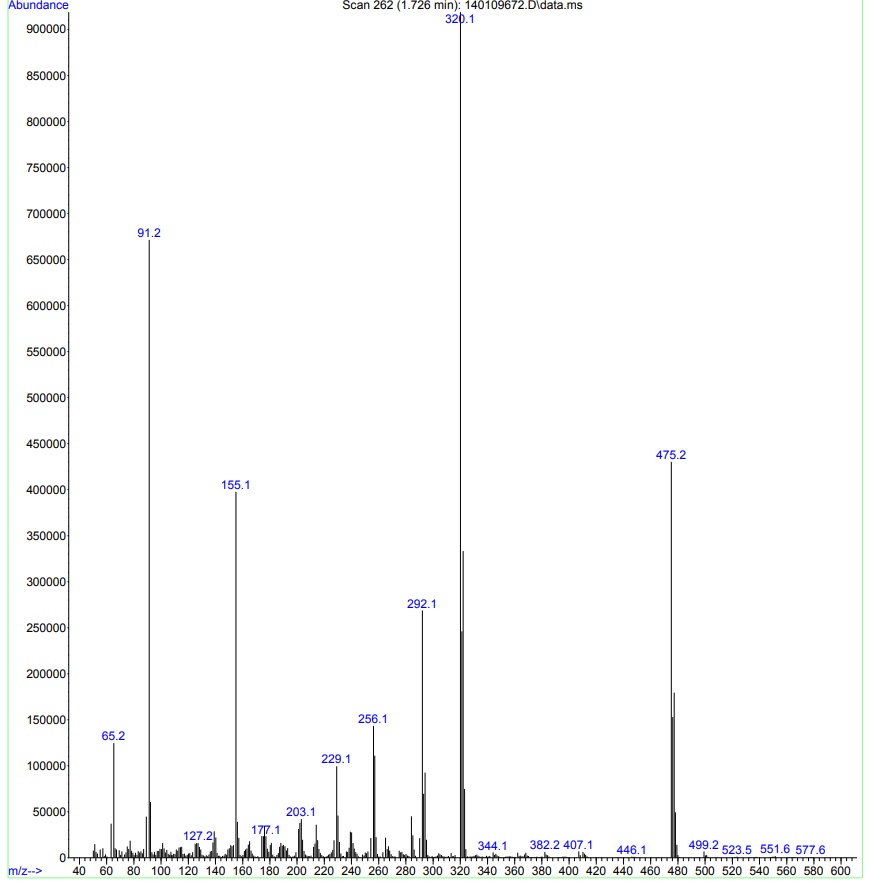


34: FT-IR spectrum of 4-(6-amino-5-cyano-4-(2,4-dichlorophenyl)pyridin-2-yl)phenyl 4-methylbenzenesulfonate. (1i)


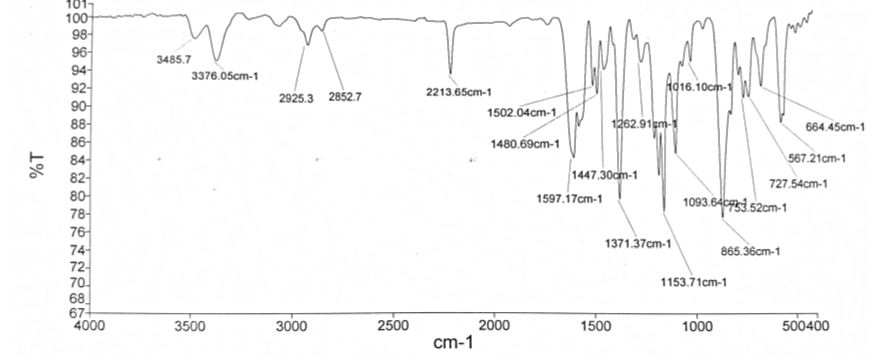


35: ^1^H NMR spectrum of 4-(6-amino-5-cyano-4-(2,4-dichlorophenyl)pyridin-2-yl)phenyl 4-methylbenzenesulfonate. (1i)


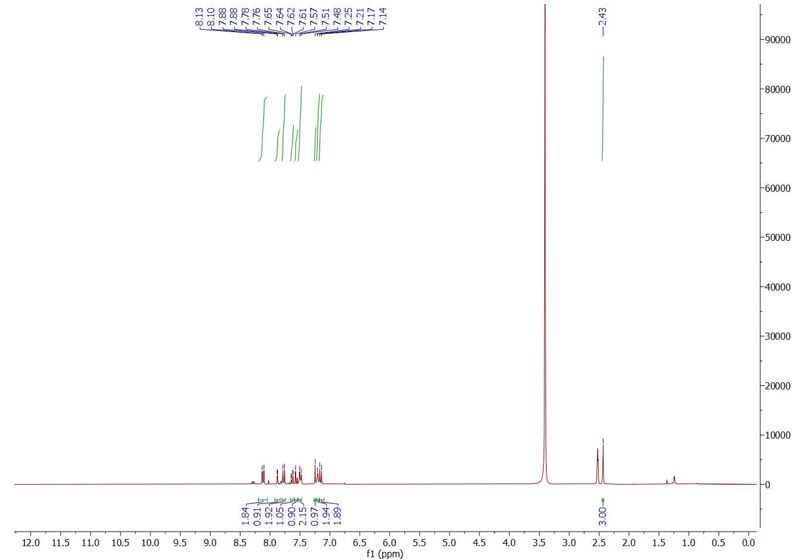


36: ^13^C NMR spectrum of 4-(6-amino-5-cyano-4-(2,4-dichlorophenyl)pyridin-2-yl)phenyl 4-methylbenzenesulfonate. (1i)
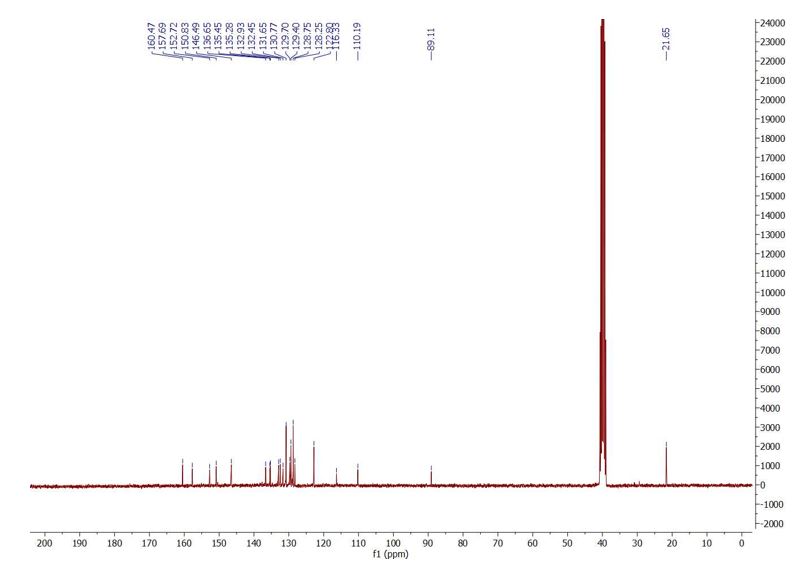


37: Mass spectrum of 4-(6-amino-5-cyano-4-(2,4-dichlorophenyl)pyridin-2-yl)phenyl 4-methylbenzenesulfonate. (1i)


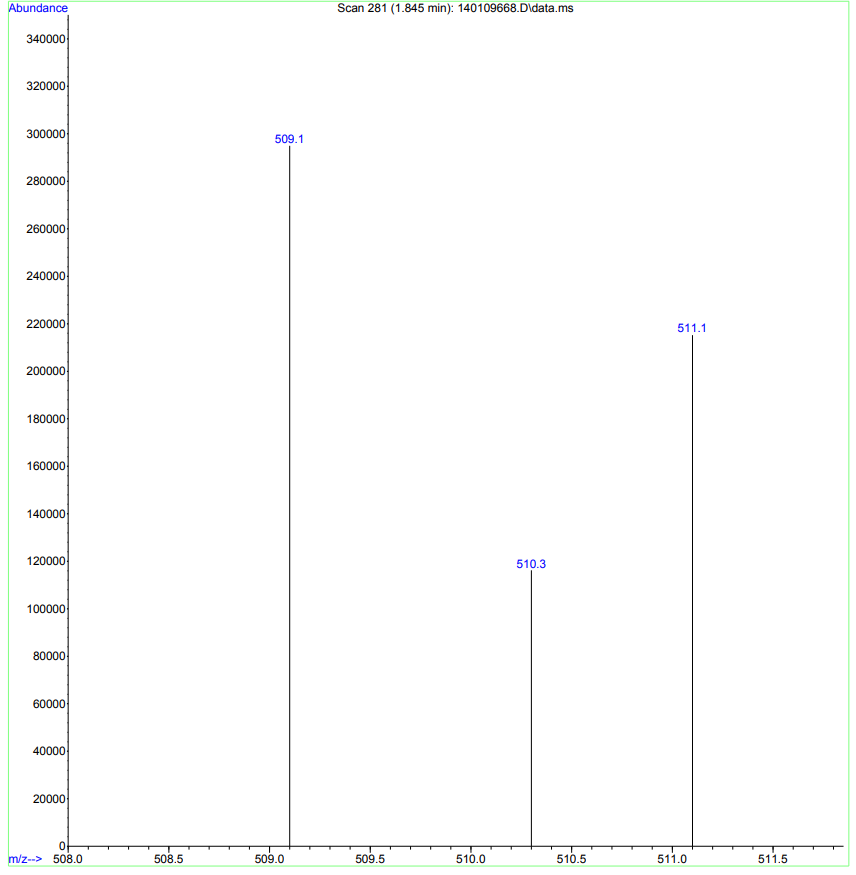


38: FT-IR spectrum of 4-(6-amino-5-cyano-4-(3-fluorophenyl)pyridin-2-yl)phenyl 4-methylbenzenesulfonate. (1j)


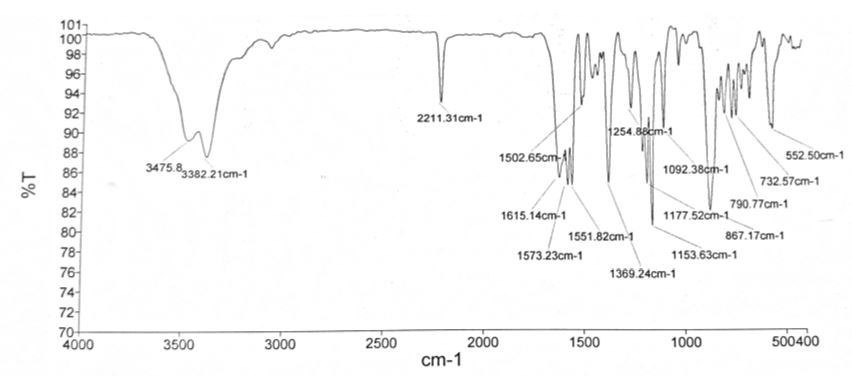


39: ^1^H NMR spectrum of 4-(6-amino-5-cyano-4-(3-fluorophenyl)pyridin-2-yl)phenyl 4-methylbenzenesulfonate. (1j)


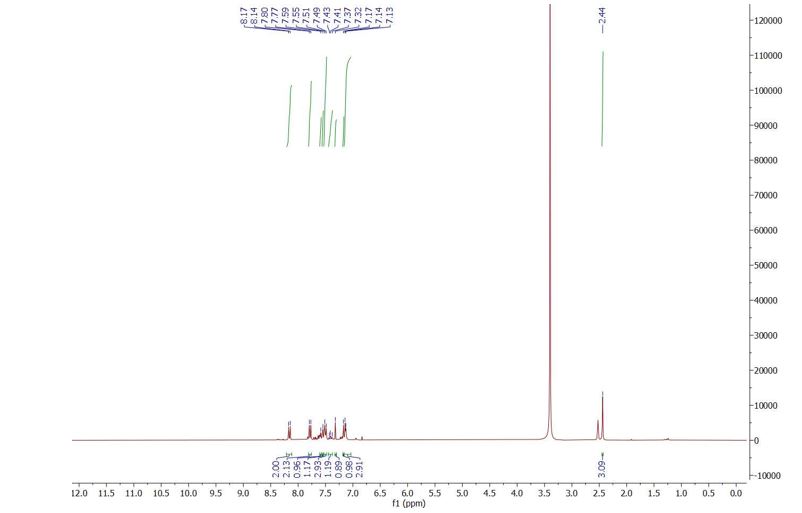


40: ^13^C NMR spectrum of 4-(6-amino-5-cyano-4-(3-fluorophenyl)pyridin-2-yl)phenyl 4-methylbenzenesulfonate. (1j)


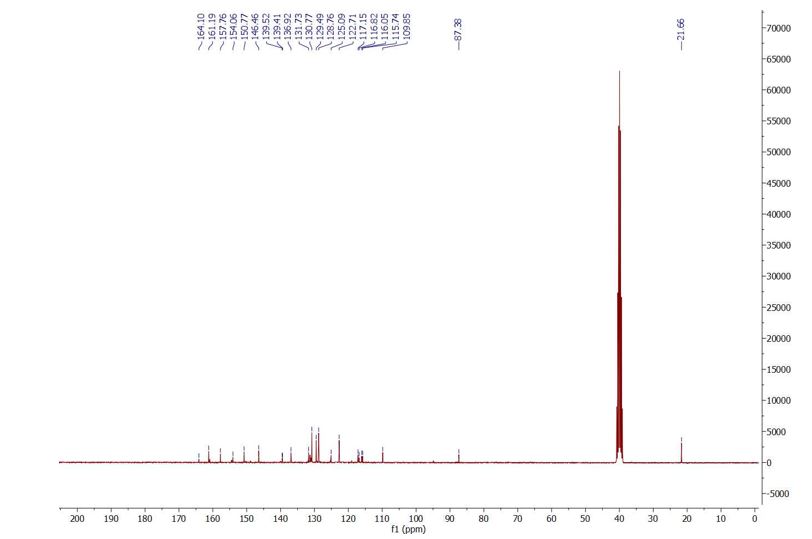


41: Mass spectrum of 4-(6-amino-5-cyano-4-(3-fluorophenyl)pyridin-2-yl)phenyl 4-methylbenzenesulfonate. (1j)


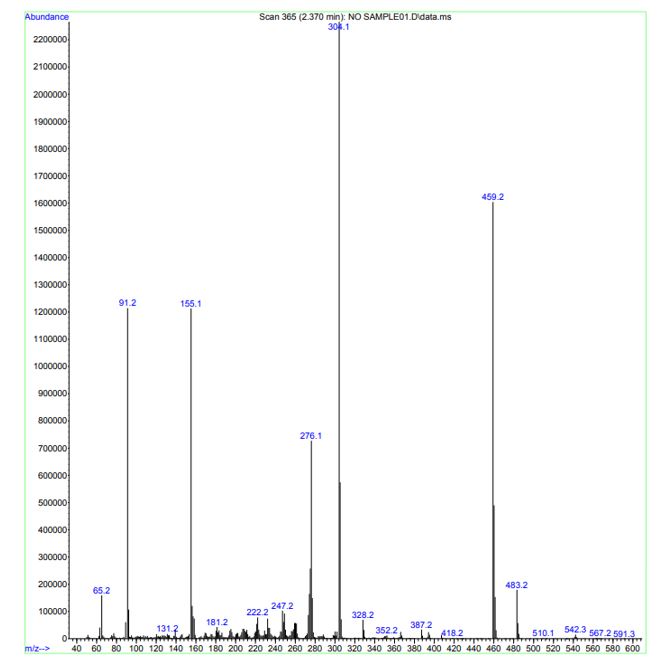


42: FT-IR spectrum of 4-(6-amino-4-(4-bromophenyl)-5-cyanopyridin-2-yl)phenyl 4-methylbenzenesulfonate. (1k)


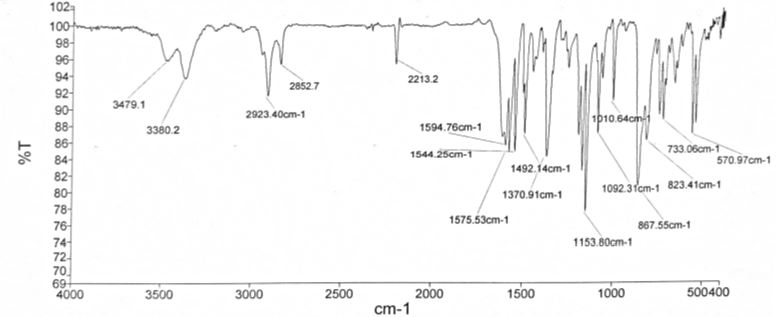


43: ^1^H NMR spectrum of 4-(6-amino-4-(4-bromophenyl)-5-cyanopyridin-2-yl)phenyl 4-methylbenzenesulfonate. (1k)


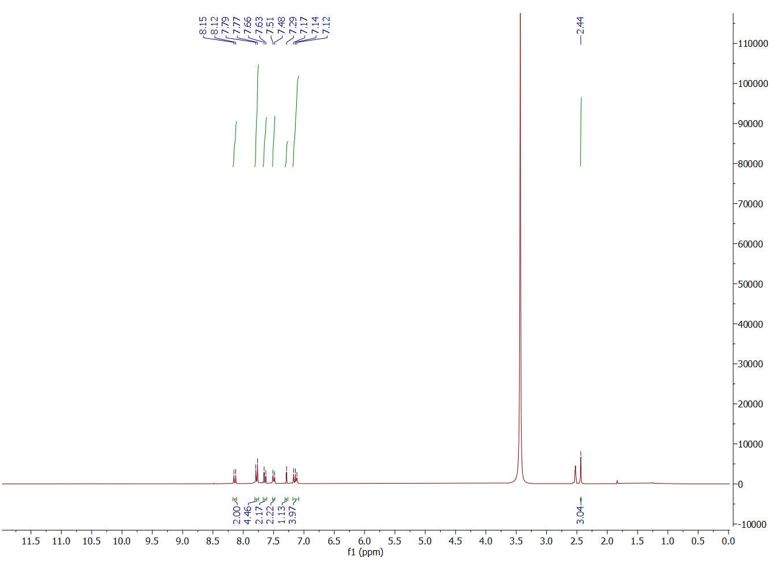


44: ^13^C NMR spectrum of 4-(6-amino-4-(4-bromophenyl)-5-cyanopyridin-2-yl)phenyl 4-methylbenzenesulfonate. (1k)


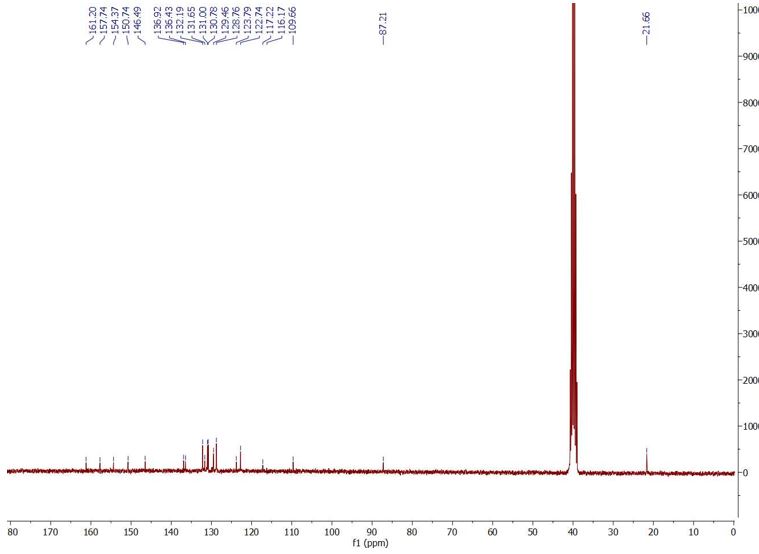


45: Mass spectrum of 4-(6-amino-4-(4-bromophenyl)-5-cyanopyridin-2-yl)phenyl 4-methylbenzenesulfonate. (1k)


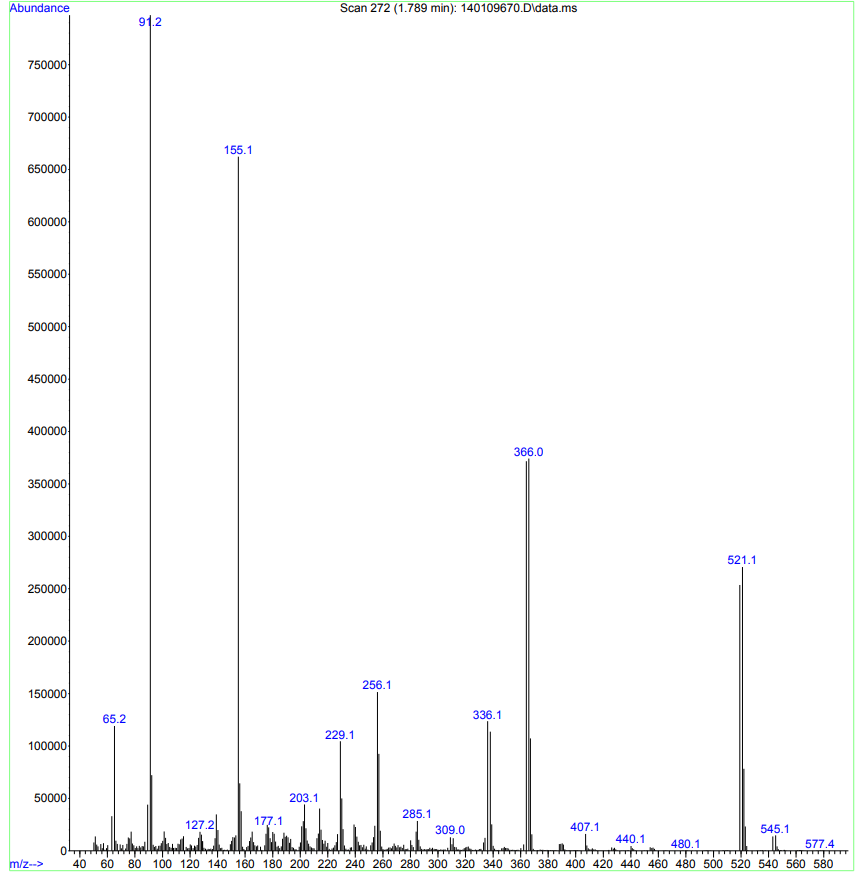


46: FT-IR spectrum of 4-(5-cyano-6-(1*H*-indol-3-yl)-4-(2-methoxyphenyl)pyridin-2-yl)phenyl 4-methylbenzenesulfonate. (2a)


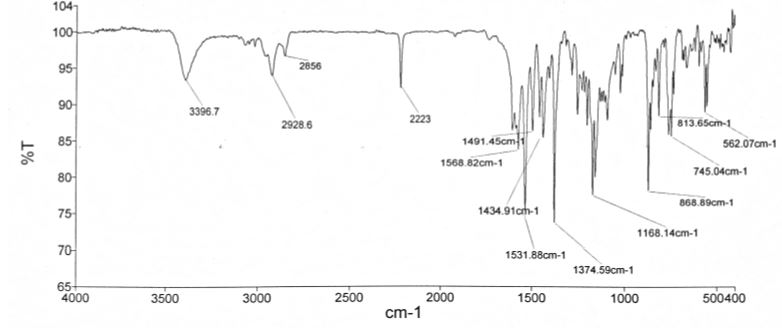


47: ^1^H NMR spectrum of 4-(5-cyano-6-(1*H*-indol-3-yl)-4-(2-methoxyphenyl)pyridin-2-yl)phenyl 4-methylbenzenesulfonate. (2a)


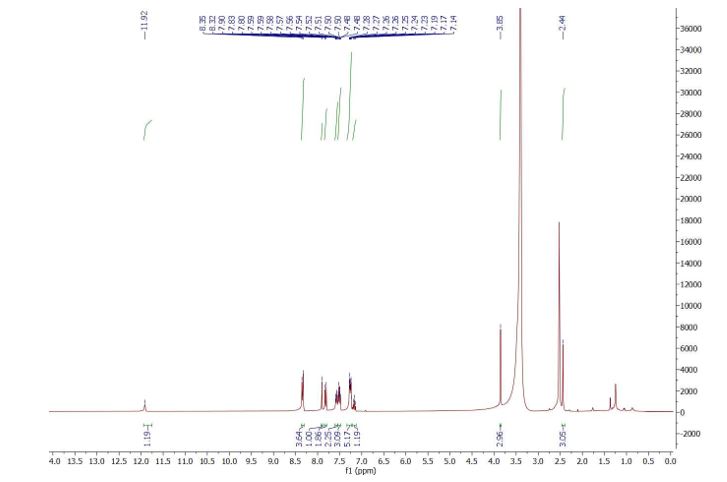


48: ^13^C NMR spectrum of 4-(5-cyano-6-(1*H*-indol-3-yl)-4-(2-methoxyphenyl)pyridin-2-yl)phenyl 4-methylbenzenesulfonate. (2a)


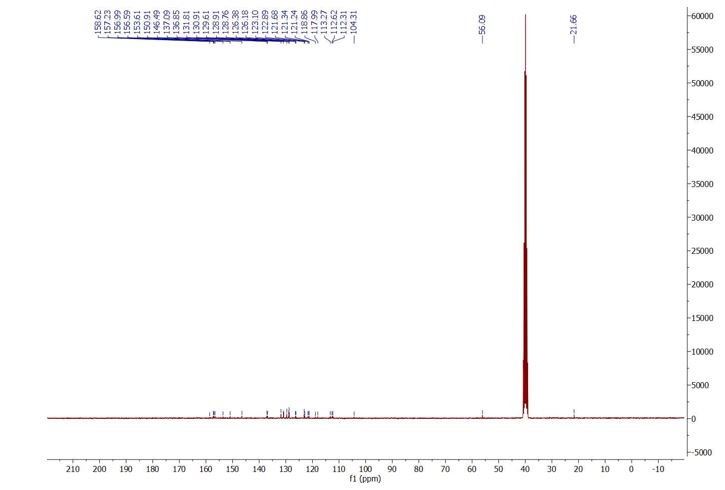


49: Mass spectrum of 4-(5-cyano-6-(1*H*-indol-3-yl)-4-(2-methoxyphenyl)pyridin-2-yl)phenyl 4-methylbenzenesulfonate. (2a)


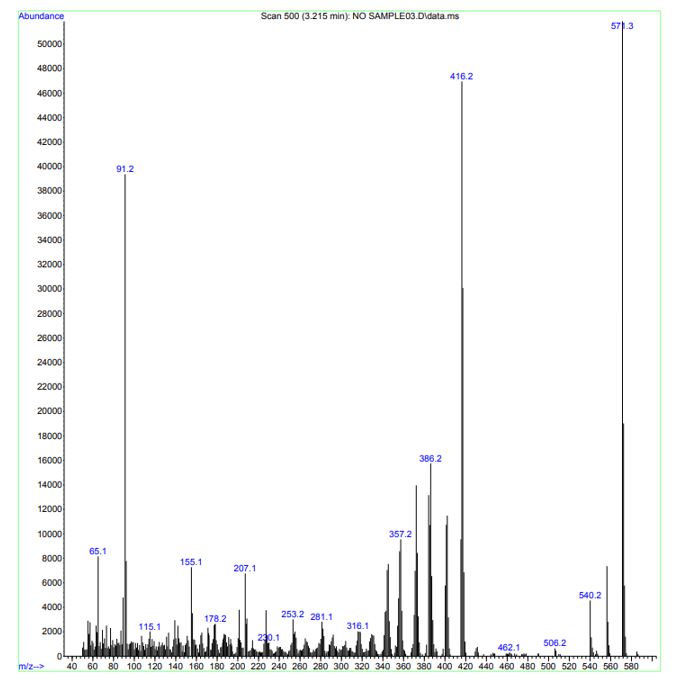


50: FT-IR spectrum of 4-(5-cyano-6-(1*H*-indol-3-yl)-4-(4-methoxyphenyl)pyridin-2-yl)phenyl 4-methylbenzenesulfonate. (2b)


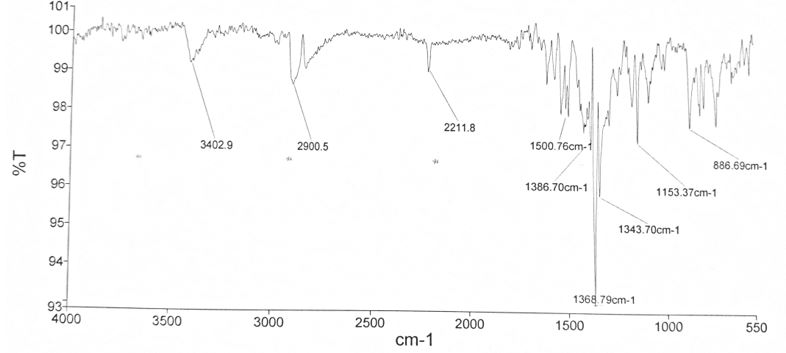


51: ^1^H NMR spectrum of 4-(5-cyano-6-(1*H*-indol-3-yl)-4-(4-methoxyphenyl)pyridin-2-yl)phenyl 4-methylbenzenesulfonate. (2b)


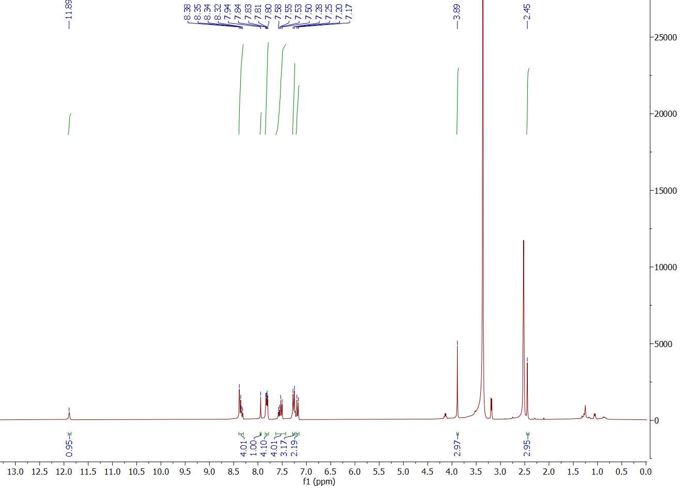


52: ^13^C NMR spectrum of 4-(5-cyano-6-(1*H*-indol-3-yl)-4-(4-methoxyphenyl)pyridin-2-yl)phenyl 4-methylbenzenesulfonate. (2b)


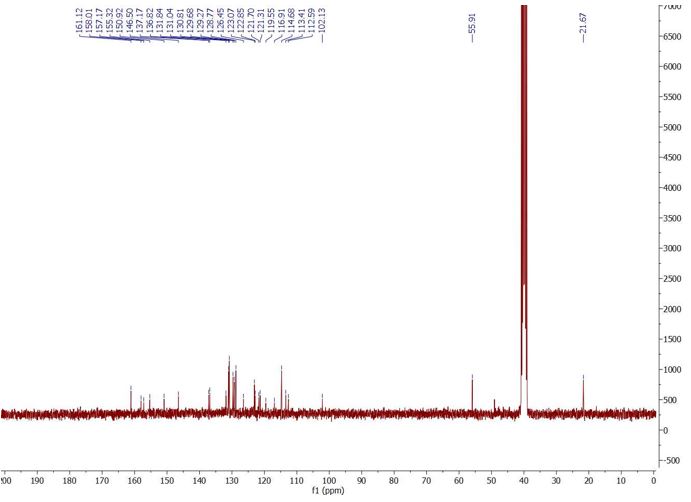


53: Mass spectrum of 4-(5-cyano-6-(1*H*-indol-3-yl)-4-(4-methoxyphenyl)pyridin-2-yl)phenyl 4-methylbenzenesulfonate. (2b)


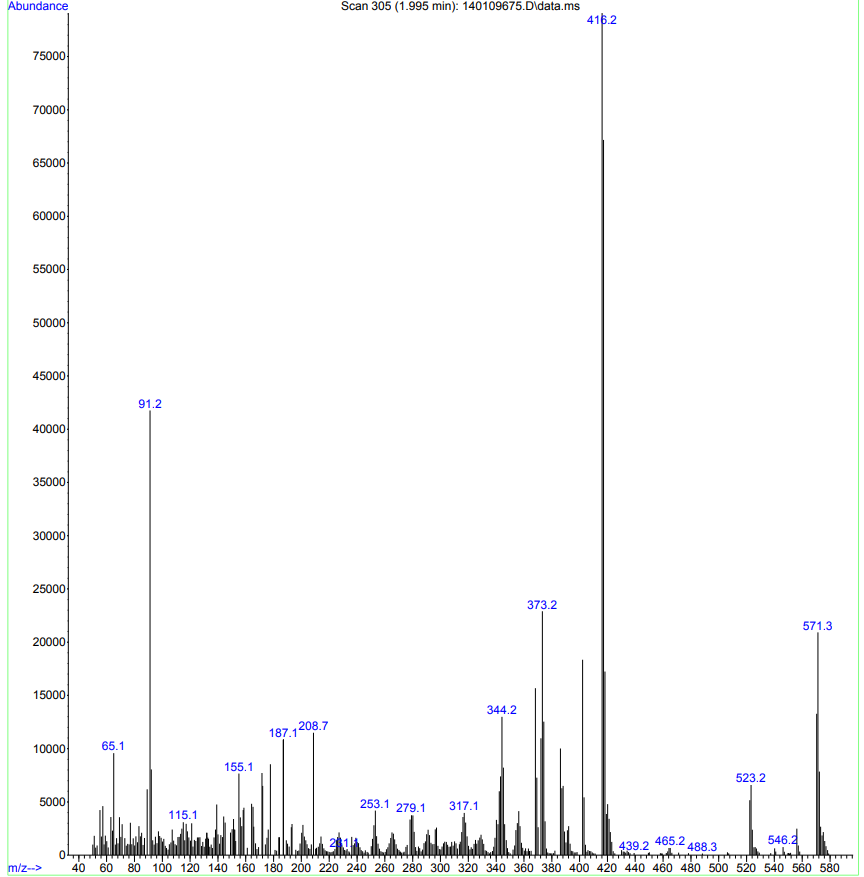


54: FT-IR spectrum of 4-(4-(4-chlorophenyl)-5-cyano-6-(1*H*-indol-3-yl)pyridin-2-yl)phenyl 4-methylbenzenesulfonate. (2c)


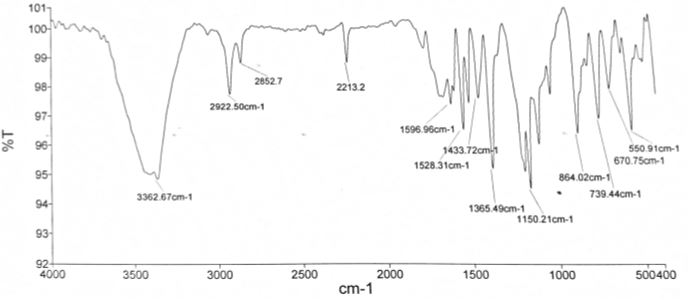


55: ^1^H NMR spectrum of 4-(4-(4-chlorophenyl)-5-cyano-6-(1*H*-indol-3-yl)pyridin-2-yl)phenyl 4-methylbenzenesulfonate. (2c)


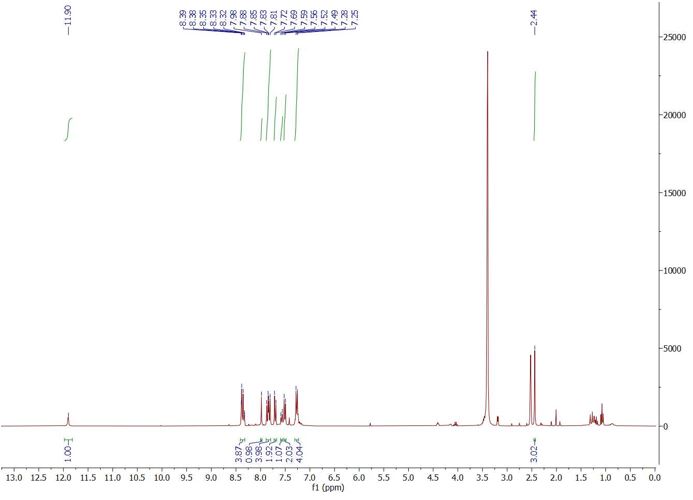


56: Mass spectrum of 4-(4-(4-chlorophenyl)-5-cyano-6-(1*H*-indol-3-yl)pyridin-2-yl)phenyl 4-methylbenzenesulfonate. (2c)


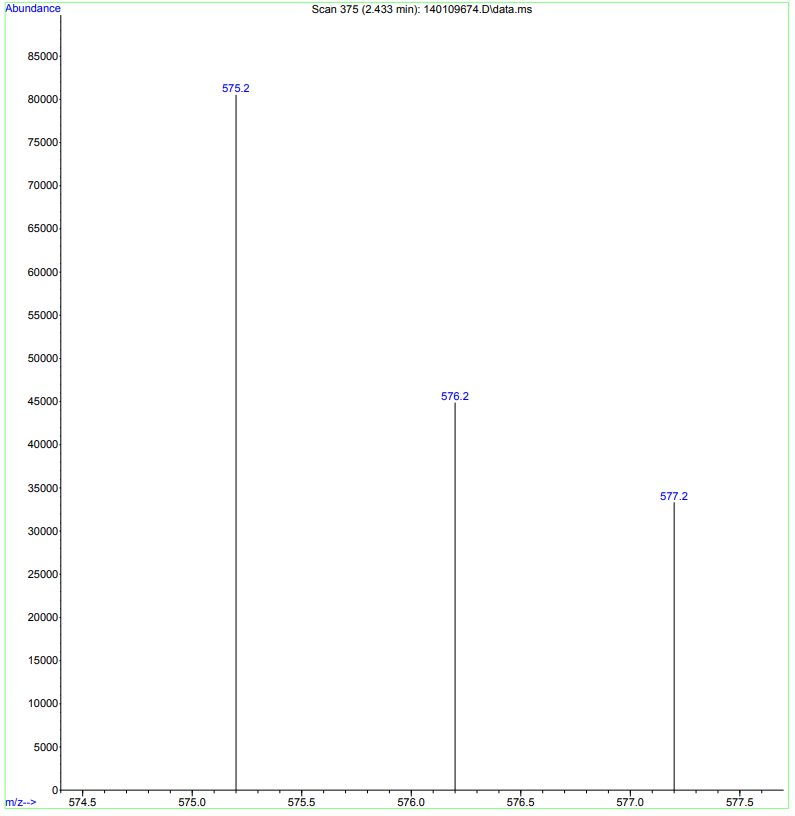


57: FT-IR spectrum of 4-(5-cyano-4-(2,4-dichlorophenyl)-6-(1*H*-indol-3-yl)pyridin-2-yl)phenyl 4-methylbenzenesulfonate. (2d)


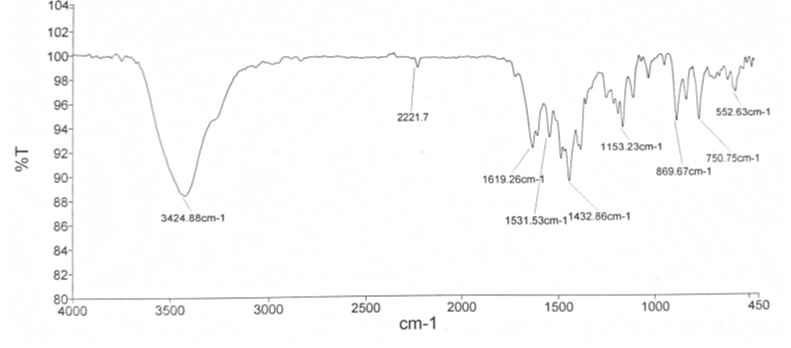


58: ^1^H NMR spectrum of 4-(5-cyano-4-(2,4-dichlorophenyl)-6-(1*H*-indol-3-yl)pyridin-2-yl)phenyl 4-methylbenzenesulfonate. (2d)


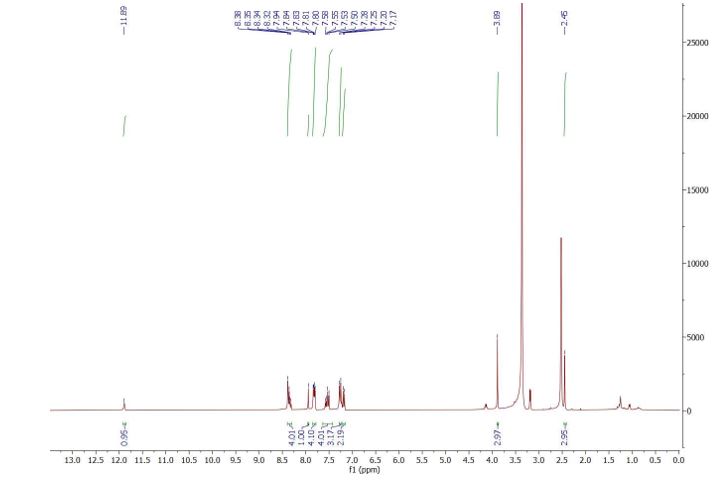


59: ^13^C NMR spectrum of 4-(5-cyano-4-(2,4-dichlorophenyl)-6-(1*H*-indol-3-yl)pyridin-2-yl)phenyl 4-methylbenzenesulfonate. (2d)


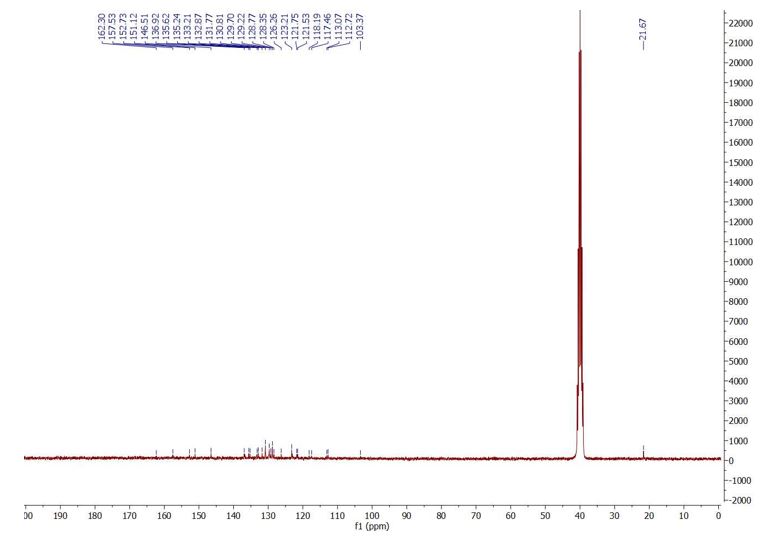


60: ^1^H NMR spectrum of 4-(4-bromophenyl)-2,6-di(1*H*-indol-3-yl) nicotinonitrile. (3a)


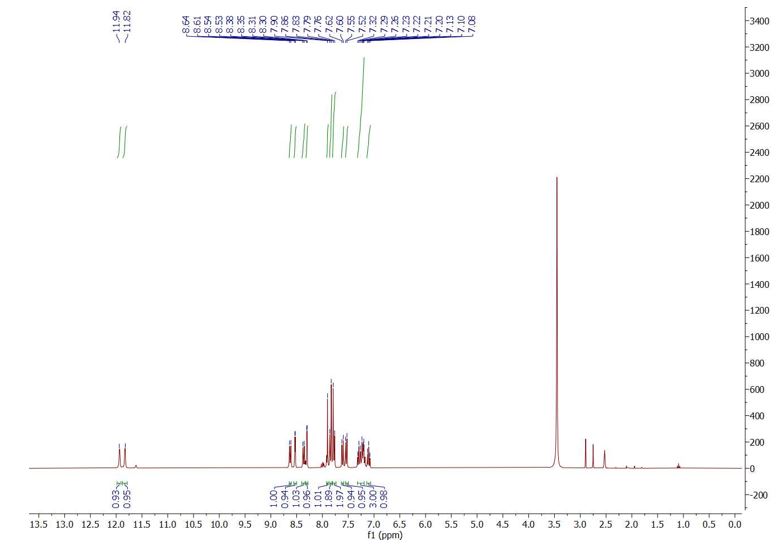


61: ^13^C NMR spectrum of 4-(4-bromophenyl)-2,6-di(1*H*-indol-3-yl) nicotinonitrile. (3a)


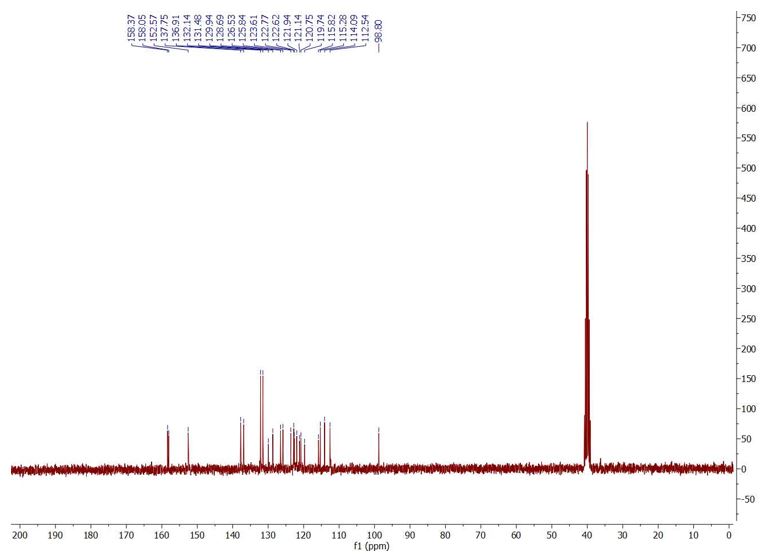


62: Mass spectrum of 4-(4-bromophenyl)-2,6-di(1*H*-indol-3-yl) nicotinonitrile. (3a)


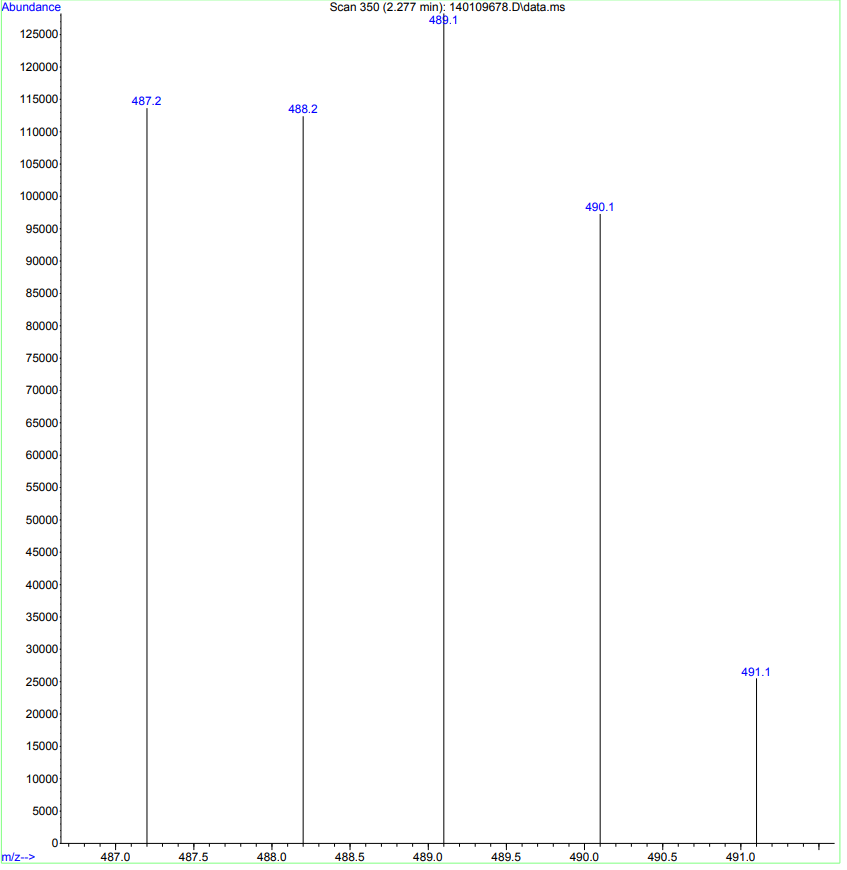


63: FT-IR spectrum of 4-(4-chlorophenyl)-2,6-di(1*H*-indol-3-yl) nicotinonitrile. (3b)


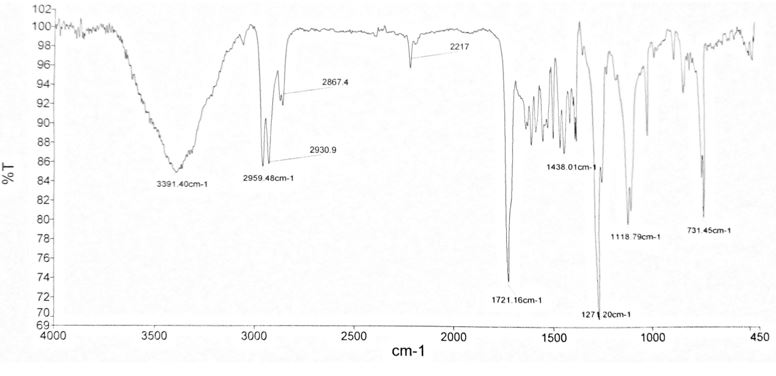


64: ^1^H NMR spectrum of 4-(4-chlorophenyl)-2,6-di(1*H*-indol-3-yl) nicotinonitrile. (3b)


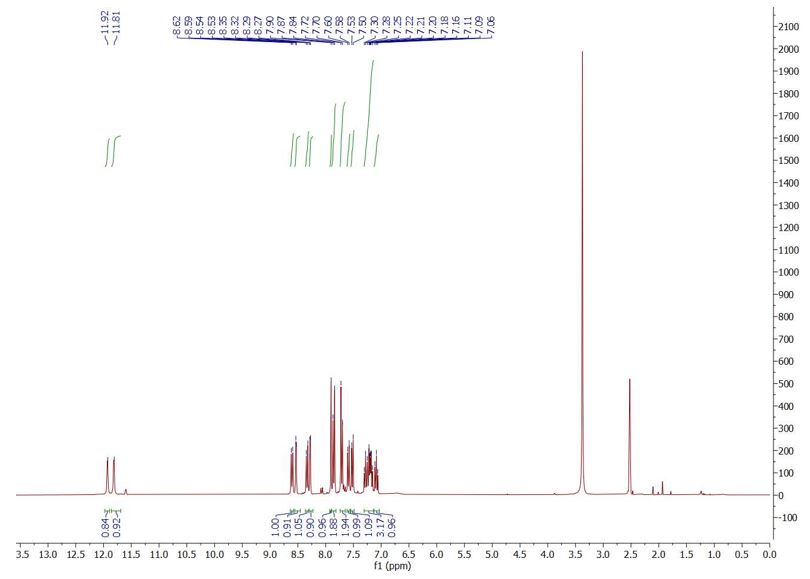


65: Mass spectrum of 4-(4-chlorophenyl)-2,6-di(1*H*-indol-3-yl) nicotinonitrile. (3b)


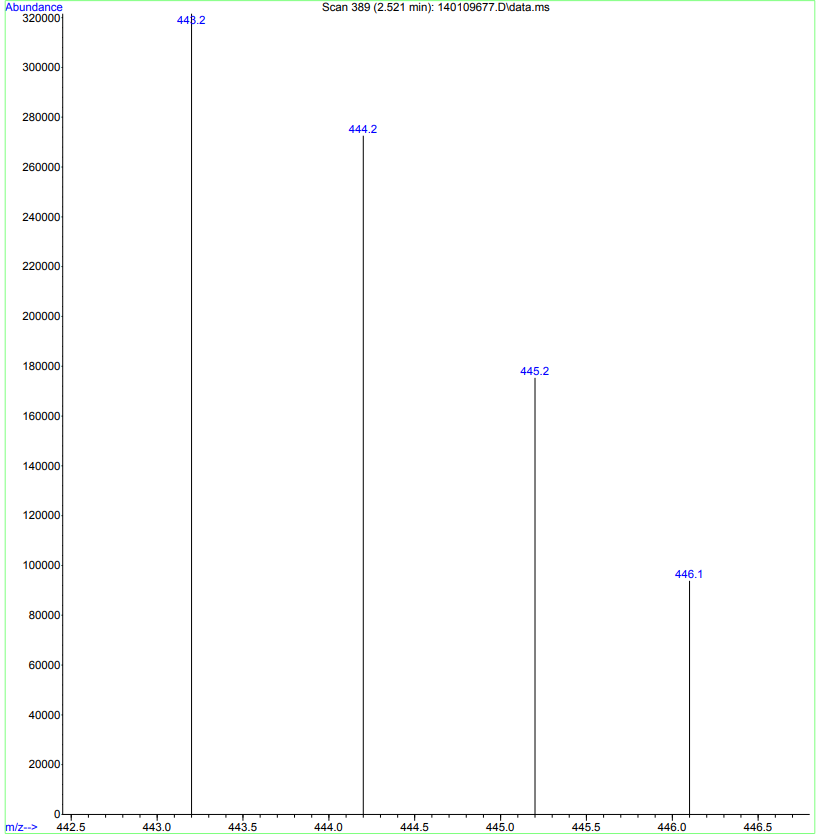


66: FT-IR spectrum of Ch-Cl/urea


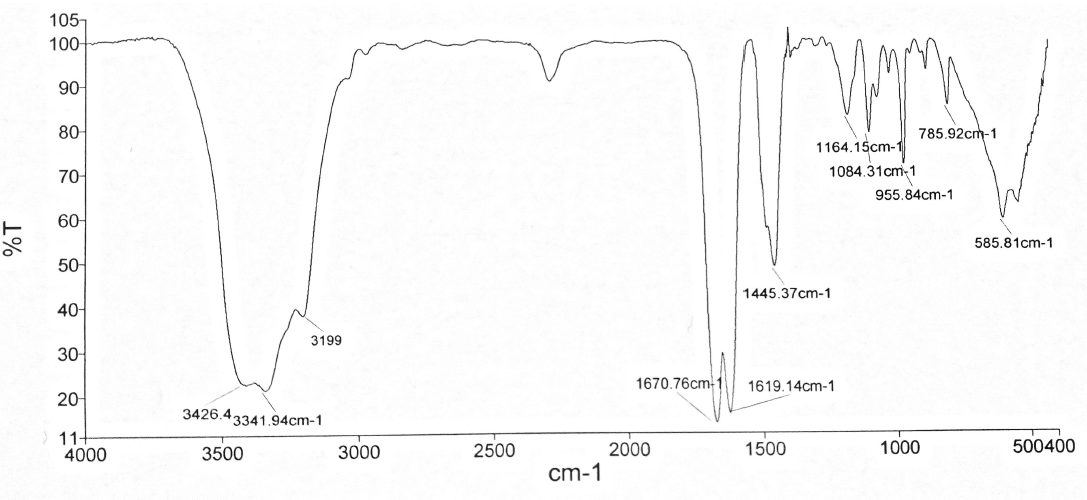


67: FT-IR spectrum of Ch-Cl/thiourea


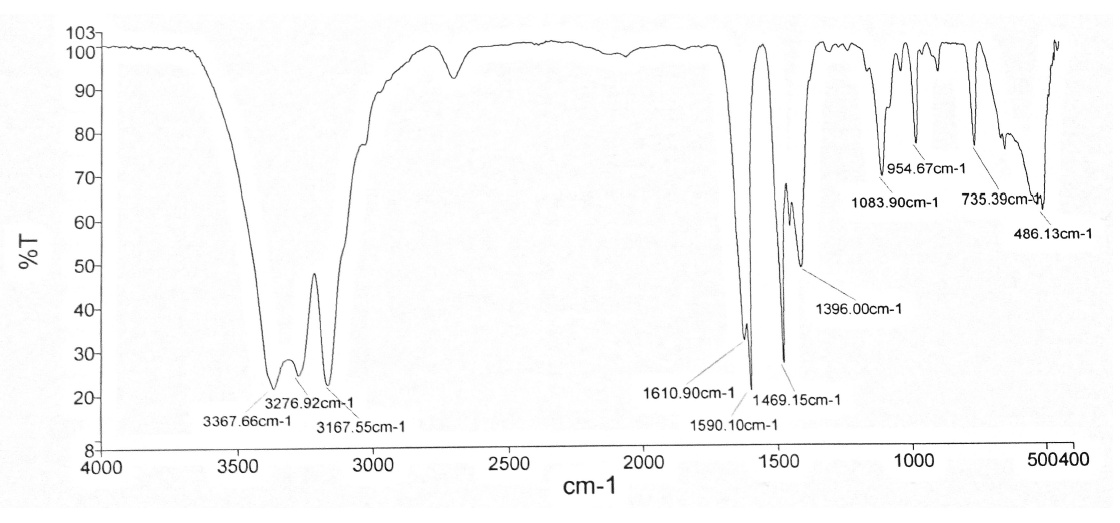


68: FT-IR spectrum of Ch-Cl/acetamide


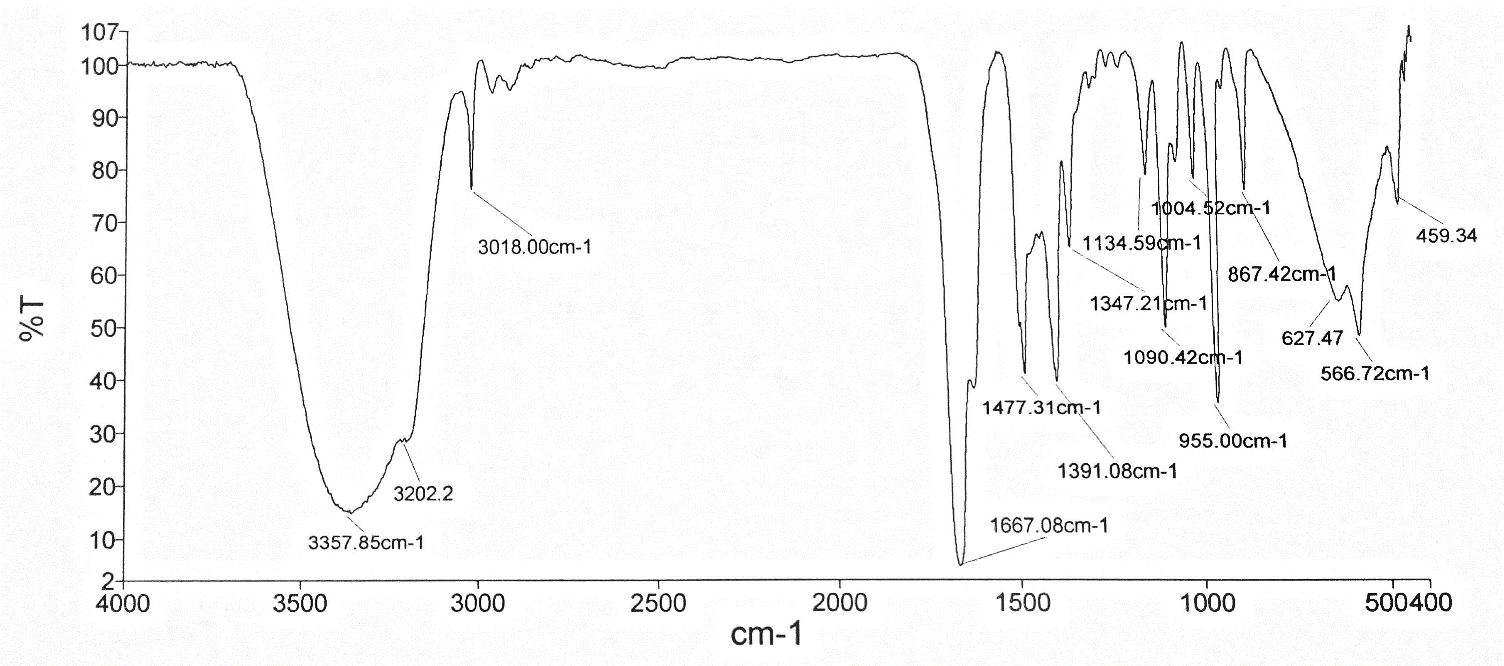


69: FT-IR spectrum of Ch-Cl/benzoic acid


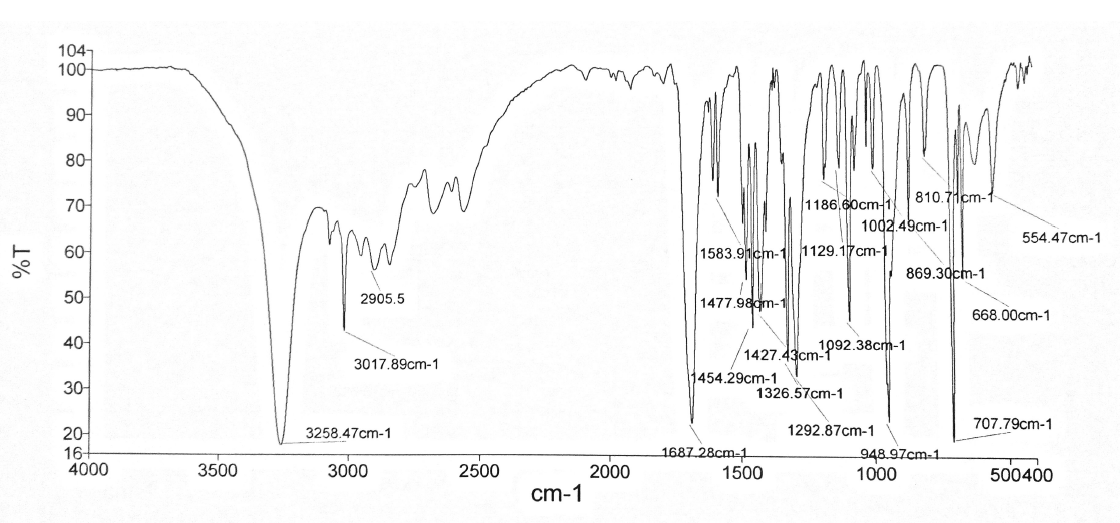


70: FT-IR spectrum of Ch-Cl/ascorbic acid


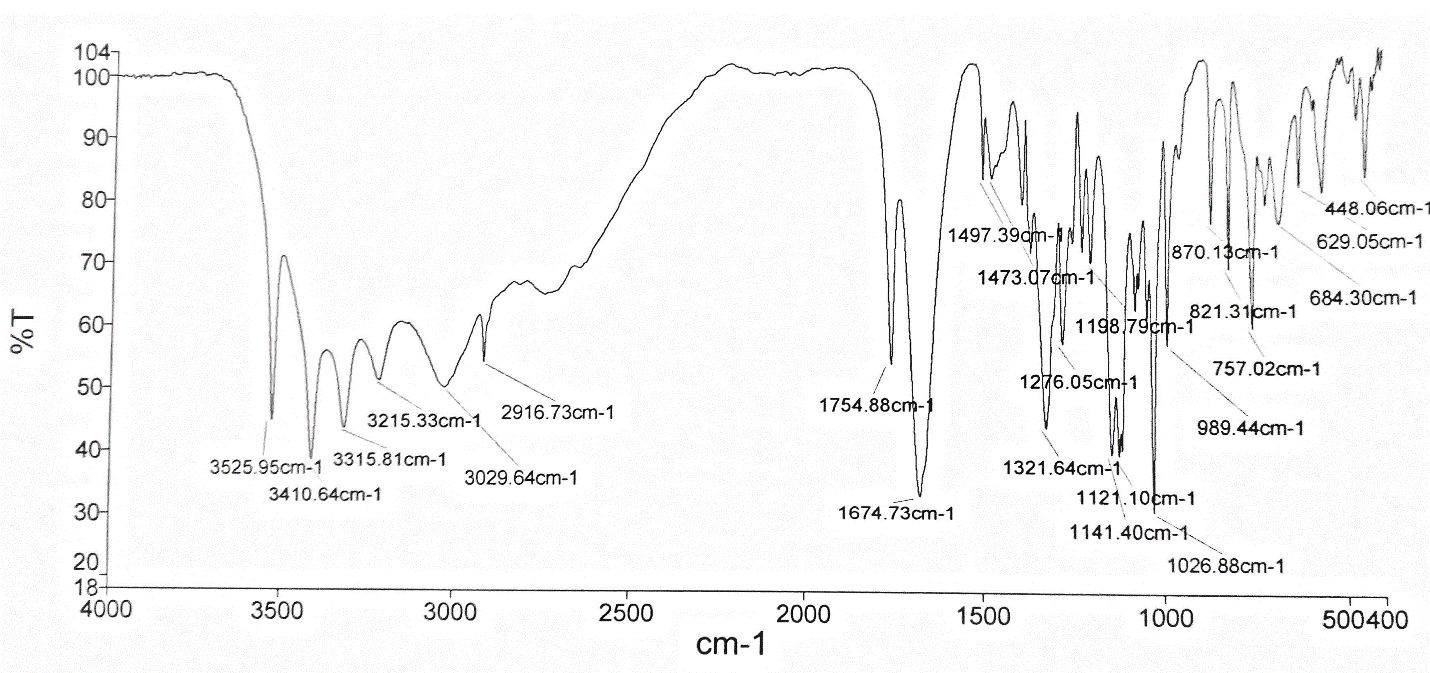

Supplement: Supplementary file 1 — Supplementary Information. [file 41598_2023_35849_MOESM1_ESM.docx]
